# Transcriptome of interstitial cells of Cajal reveals unique and selective gene signatures

**DOI:** 10.1371/journal.pone.0176031

**Published:** 2017-04-20

**Authors:** Moon Young Lee, Se Eun Ha, Chanjae Park, Paul J. Park, Robert Fuchs, Lai Wei, Brian G. Jorgensen, Doug Redelman, Sean M. Ward, Kenton M. Sanders, Seungil Ro

**Affiliations:** 1 Department of Physiology and Cell Biology, University of Nevada School of Medicine, Reno, Nevada, United States of America; 2 Department of Physiology, Wonkwang Digestive Disease Research Institute and Institute of Wonkwang Medical Science, School of Medicine, Wonkwang University, Iksan, Jeollabuk-do, Korea; University of Texas Medical Branch, UNITED STATES

## Abstract

Transcriptome-scale data can reveal essential clues into understanding the underlying molecular mechanisms behind specific cellular functions and biological processes. Transcriptomics is a continually growing field of research utilized in biomarker discovery. The transcriptomic profile of interstitial cells of Cajal (ICC), which serve as slow-wave electrical pacemakers for gastrointestinal (GI) smooth muscle, has yet to be uncovered. Using copGFP-labeled ICC mice and flow cytometry, we isolated ICC populations from the murine small intestine and colon and obtained their transcriptomes. In analyzing the transcriptome, we identified a unique set of ICC-restricted markers including transcription factors, epigenetic enzymes/regulators, growth factors, receptors, protein kinases/phosphatases, and ion channels/transporters. This analysis provides new and unique insights into the cellular and biological functions of ICC in GI physiology. Additionally, we constructed an interactive ICC genome browser (http://med.unr.edu/physio/transcriptome) based on the UCSC genome database. To our knowledge, this is the first online resource that provides a comprehensive library of all known genetic transcripts expressed in primary ICC. Our genome browser offers a new perspective into the alternative expression of genes in ICC and provides a valuable reference for future functional studies.

## Introduction

Interstitial cells of Cajal (ICC) are pacemaker cells that respond to enteric motor neurotransmitters in the gastrointestinal (GI) tract [1]. Networks of ICC are widely distributed and display a range of different morphologies in different parts of the GI tract. ICC subtypes are named based on their locations within tissues. A consistent biomarker for ICC is expression of *Kit*. This gene encodes a receptor tyrosine kinase, KIT (aka c-kit), that binds stem cell factor (SCF or KITL). SCF/KIT signaling is required for the normal development and maintenance of ICC in the GI tract [2, 3]. Inhibition of, and loss-of-function mutations in, *Kit* caused the loss of ICC in GI muscles [2, 4–6], and loss of ICC and/or defects in the continuity of ICC networks have been associated with GI motility disorders in both animals and humans [2, 7].

Many studies of ICC utilized KIT antibodies (aka anti-CD117) to identify these cells in tissues and in cultures [8–12]. While immuno-localization has been shown to be useful, detection of ICC within fresh dispersions of GI muscles was limited. These problems were overcome through the development of *Kit*^*+/copGFP*^ mice in which all subtypes of ICC were labeled constitutively with the reporter, copGFP [13]. With this tool, we found that ICC express the gene, anoctamin 1 (*Ano1*; formerly known as *Tmem16a*) that encodes a Ca^2+^-activated Cl^-^ channel that is involved in pacemaker activity and generation of electrical slow waves in GI muscles [14, 15]. Despite knowing that ICC express these specific biomarkers, a comprehensive reference of genome-wide transcripts (transcriptome) expressed within these cells is still elusive.

We have previously isolated GFP-labeled smooth muscle cells (SMC) from the jejunum and colon of *smMHC*^*Cre-eGFP/+*^ mice and identified genome-scale gene expression data from these cells, as well as built a SMC genome browser [16] linked to the bioinformatics data repository found in the University of California, Santa Cruz (UCSC) genome database [17]. In the present study, we used a similar strategy to isolate ICC from *Kit*^*+/copGFP*^ mice and used RNA-seq techniques to sequence the transcriptomes of ICC from the murine jejunum and colon. This information was incorporated into the UCSC Smooth Muscle Genome Browser. In analyzing the transcriptome, we identified new selective markers for ICC: thrombospondin-4 (*Thbs4*) and hyperpolarization-activated cyclic nucleotide-gated K^+^ channel 4 (*Hcn4*). We also identified several additional ion channels and transporters (*Cacng6*, *Cacng8*, *Cacnb4*, *Kcng3*, *Abcc8*, *Kcnkj2*, *Kcnmb2*, and *Slc4a4*) that are characteristic of ICC cellular identity and function. The ICC genome browser will serve as a reference, providing information about the possible structure, isoforms, and regulation of all genes expressed in ICC.

## Materials and methods

### Animal and tissue preparation

The *tunica muscularis* from the murine jejunum/colon was obtained and isolated from *Kit*^*+/copGFP*^ mice that we have previously generated [13]. These tissues were then used to isolate ICC through flow cytometry. Our animal protocol was approved by the Institutional Animal Care and Use Committee at the University of Nevada-Reno (UNR). UNR is fully accredited by AAALAC International. The colony of laboratory mice included in this experiment were housed in a Centralized Animal Facility at the UNR Animal Resources. Animals were euthanized by CO_2_ inhalation overdose in accordance with the 2013 guidelines by the American Veterinary Medical Association.

### Flow cytometry and fluorescence-activated cell sorting (FACS)

Cells were dispersed from jejunal and colonic *tunica muscularis*, and copGFP^+^ ICC were sorted from dispersed cells using FACS as described [13]. Isolated ICC were pooled from approximately 40 mice (20 males and 20 females) which were used as one collective sample in the isolation of total RNAs.

### Isolation of total RNAs

Total RNA was isolated from jejunal ICC (JICC), and colonic ICC (CICC) using the mirVana miRNA isolation kit (Life Technologies, Carlsbad, CA). The quality of total RNAs was analyzed using a NanoDrop 2000 Spectrometer (Thermo Scientific, Waltham, MA) and a 2100 Bioanalyzer (Agilent Technologies, Santa Clara, CA).

### Real time PCR

The cDNA libraries were constructed through reverse transcription of total RNAs isolated from FACS-purified ICC as previously described [13]. RT-PCR and qPCR analyses of cDNA were performed as previously described [13, 18]. All primers used for RT-PCR and qPCR are shown in S11 Table.

### Construction of RNA-seq libraries and next-generation sequencing

Two RNA-seq libraries were generated and sequenced via Illumina HiSeq 2000 (Illumina, San Diego, CA) following the vendor’s instruction at LC Sciences (Houston, TX) as previously described [16].

### Bioinformatics data analysis

Paired-end sequencing reads were processed and analyzed as described [16]. A cutoff of FPKM = 0.025 generated equal false positive and false negative ratios of reliability. The expression levels of transcripts with FPKM values less than 0.025 was considered to be 0.

### Confocal microscopy and immunohistochemical analysis

Microscopic analysis of copGFP fluorescence within the jejunum and colon was performed as previously described [13]. Jejunal and colonic tissue was analyzed by whole mount and cryostat section staining using confocal microscopy as previously described [19]. Primary antibodies against the following antigens were used: anti-ANO1 (mouse, 1:200, Abcam, Cambridge, MA), anti-KIT (goat, 1:20, R&D Systems, MN), anti-THBS4 (rabbit, 1:100, Santa Cruz, CA), and anti-HCN4 (rabbit, 1:100, alomone labs, Jerusalem, Israel). Images were collected using the Fluoview FV10-ASW 3.1 Viewer software (Olympus, Tokyo, Japan) with an Olympus FV1000 confocal laser scanning microscope.

### Western blot

Protein was extracted from *tunica muscularis* samples from *Kit*^*+/copGFP*^ mice and Western blotting was performed as previously described [20]. Primary antibodies against the following antigens were used: THBS4 (rabbit, 1:1000, Abcam, Cambridge, MA), ANO1 (rabbit, 1:000, Abcam, Cambridge, MA), HCN4 (rabbit, 1:500, alomone labs, Jerusalem, Israel), or GAPDH (rabbit, 1:2000, Cell Signaling, MA).

### Availability of supporting data

The ICC transcriptome was added to the Smooth Muscle Genome Browser [16] in the custom track of the UCSC genome database [17]. The UCSC Smooth Muscle Genome Browser is available at http://med.unr.edu/physio/transcriptome (requires Google Chrome and takes ~1 minutes to upload the large files). The genome browser contains the transcriptome menus on the “Custom Tracks.” Each menu has different display options.

The abbreviated instructions are as follows: 1) To search transcriptional variants of a gene, type in the gene symbol, and click “go.” 2) Under “Custom Tracks,” select the view option (e.g., “full”) for type of sample (e.g., “ICC Jejunum”), and click “refresh.” 3) Select the bioinformatics data of interest (e.g., click on “full” under “RefSeq Genes” in “Genes and Gene Predictions”), and then click “refresh.” 4) Click “configure” to optimize views (change image width and text size).

The RNA-seq data from this study have been also deposited in the NCBI: jejunal ICC, GSM1388408 and colonic ICC, GSM1388409.

## Results

### Identification and isolation of mature ICC

**Fig 1:**
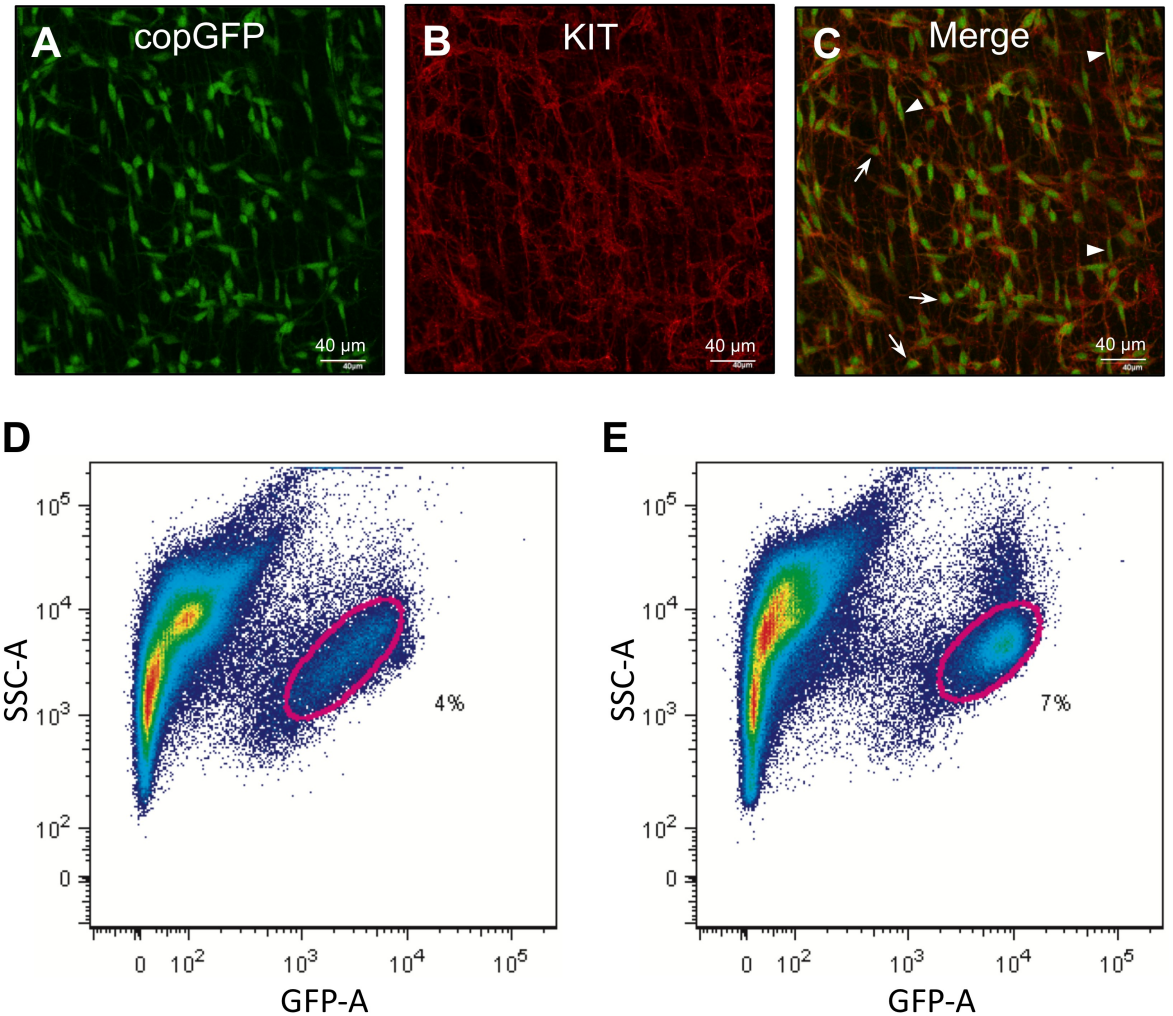
Identification of ICC in intestinal smooth muscle with copGFP and KIT antibody. (A) A z-stack image, obtained through confocal microscopy, of whole-mount jejunum muscularis showing ICC expressing copGFP. (B) Immunohistochemistry of ICC using anti-KIT antibody. (C) Merged images of copGFP and KIT showing ICC-MY (arrows) and ICC-DMP (arrow heads). (D and E) Primary copGFP^+^ ICC from jejunum and colon identified (circled) on flow cytometry.

CopGFP-labeled cells within jejunal smooth muscle layers were identified by confocal microscopy (Fig 1A), and these cells were confirmed to be Kit^+^ ICC by immunohistochemical labeling with KIT antibodies (Fig 1B), as previously reported [13]. ICC within the plane of the myenteric plexus (ICC-MY) and the deep muscular plexus (ICC-DMP) were labeled by copGFP (labeling of cytoplasm) and KIT antibodies (labeling of plasma membrane) (Fig 1C). ICC were enzymatically isolated from the jejunum and colon and sorted to purity by FACS. Cells with copGFP from the jejunum and colon were identified microscopically after sorting (Fig 1D and 1E). Since a limited number of isolated ICC were obtained from each tissue sample, jejunal and colonic ICC from 40 mice were sorted and pooled together for mRNA isolation and genetic analysis.

### Comparison and analysis of ICC transcriptomes in jejunum and colon

**Fig 2:**
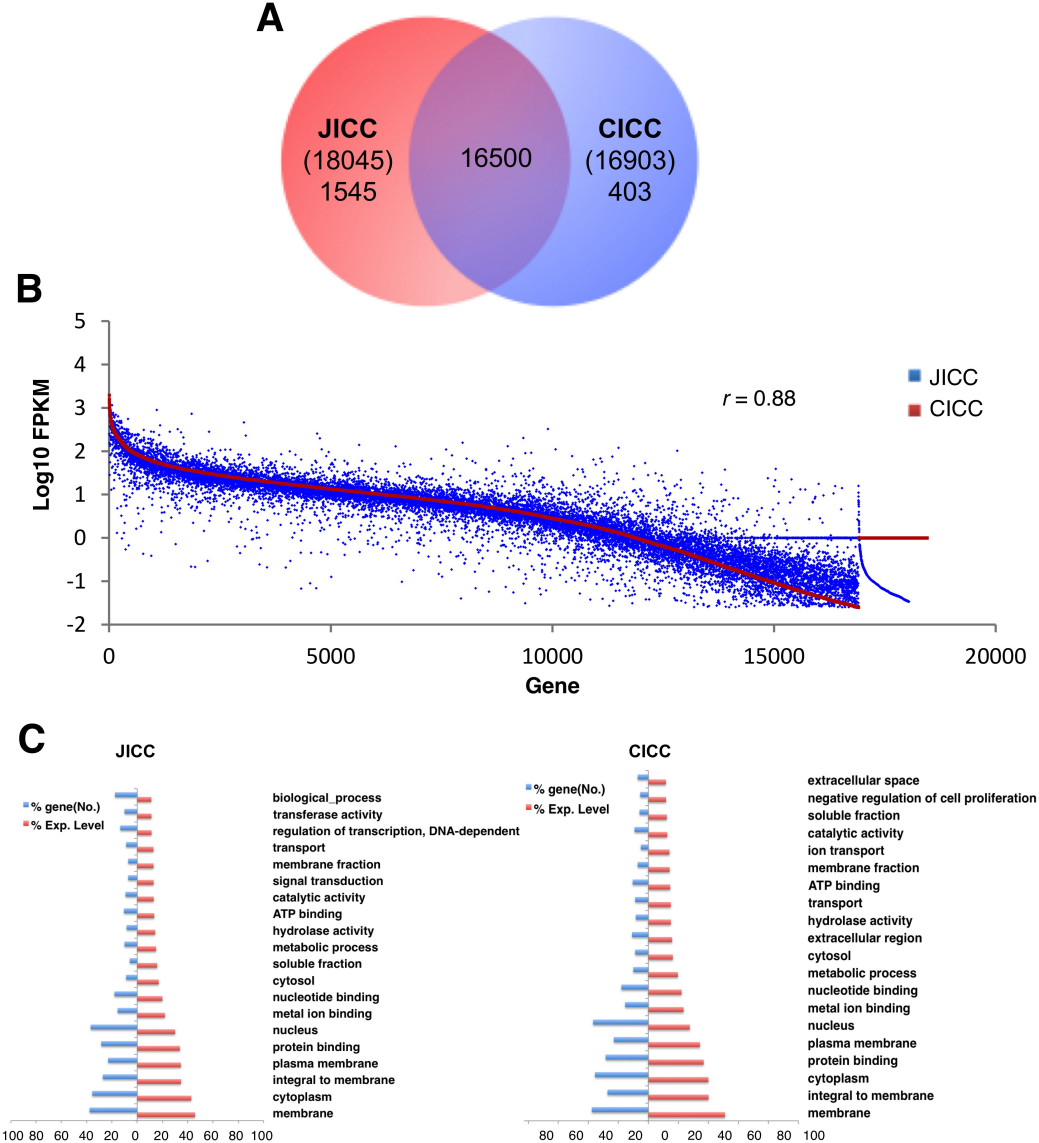
Transcriptome comparison between jejunal and colonic ICC. (A) Venn diagram showing the total number of genes expressed in JICC and CICC. (B) Comparison of expression levels of genes in JICC and CICC. (C) Gene ontologies reported in JICC and CICC. A gene ontology analysis (GO: function, process, and component) of ICC-specific genes was performed, and key GO terms were compared with a normalized expression percentile (*left*, number of genes; *right*, expression level of genes).

To identify genes expressed in ICC, RNA-seq was performed on mRNA samples from sorted jejunal (JICC) and colonic ICC (CICC). We obtained 206.7 million reads from JICC and 193.2 million read from CICC, of which, 86%-87% were successfully mapped onto the genome. The transcriptomes of JICC and CICC included 18,045 and 16,903 known genes, respectively (S1 Table). We identified 55,259 and 54,488 gene isoforms from JICC and CICC, respectively. The complete list of all isoforms identified, along with their respective tracking ID, gene ID/name, chromosome location, isoform length, and expression levels found within JICC and CICC are found in S2 Table. ICC expressed an average of three isoforms per gene (S1 Table), which was the result of alternative transcription start sites and/or post-transcriptional alternative splicing. Most genes (91–98%) were expressed in JICC and CICC, but approximately 2,000 genes were determined to be organ-specific (Fig 2A). A complete list of all genes expressed in JICC and CICC, as well as their combined expression levels from all of the transcriptional variants, are shown in S3 Table.

We also quantified the expression levels of all known genes expressed in ICC. While several hundred highly expressed genes were found in both JICC and CICC, genes with lower expression generally had a higher discrepancy in levels of expression between JICC and CICC (Fig 2B). The overall expression profiles in ICC from the jejunum and the colon produced a correlation coefficient of 0.88, indicating a high degree of similarity between the cells from the two separate tissues. The expression levels of several cell-specific markers for cells found in GI tissue (*Kit* for ICC, *Myh11* for SMC, *Pdgfra* for PDGFRα^+^ cells, and *Uchl1* [aka PGP9.5] for neuronal cells) were quantified to further validate the identity of the ICC obtained after FACS. As expected, *Kit* expression was far higher in JICC and CICC, as compared to expression levels in jejunal and colonic tissue (S1A Fig). Additionally, expression levels of *Myh11* and *Uchl1* were minimal in ICC, in comparison to jejunal and colonic tissue (S1B and S1D Fig). However, *Pdgfra* was noticeably expressed in CICC, with a lower level of expression found in JICC (S1C Fig). Since colonic ICC and PDGFRα^+^ cells are closely associated in the same anatomical locations (e.g. within muscle bundles and between the circular and longitudinal muscle layers) [21,22], CICC may have been contaminated with PDGFRα^+^ cells. To see if *Pdgfra* expression in CICC was due to a contamination of PDGFRα^+^ cells, we examined if other markers of PDGFRα^+^ cells were also expressed in CICC. PDGFRα (*Pdgfra*) and PDGFRβ (*Pdgfrb)* form either a homodimeric or heterodimeric receptor [23]. PDGFRα^+^ cells also specifically express *Kcnn3* (SK3 channel) [24], *P2ry1* [25], and *Cd34* [24] in the colonic smooth muscle. Furthermore, we found that these cells express *Ces1d* in a PDGFRα^+^ cell-specific manner (unpublished data). In our transcriptome data, CICC expressed *Pdgfra* and *Pdgfrb*, but not or little expressed *Kcnn3*, *P2ry1*, *Cd34*, and *Ces1d* (S1E Fig). These six genes were predominantly expressed in PDGFRα^+^ cells compared to whole colon muscle tissue. This expression data suggests that the expression of *Pdgfra* and *Pdgfrb* in CICC was not artificial, but specific to the cells.

Further investigation of the transcriptome data revealed how genes expressed by ICC fit into defined gene ontology (GO) groups relating to the known physiological functions of ICC (Fig 2C). GO classifications shared by jejunal and colonic ICC were multiple membrane related categories, including an enrichment of genes involved in ion transport and metal ion binding (Fig 2C), which may be important in setting membrane potentials and the generation of pacemaker activity. The similarities in GO terms found in the transcriptomes of ICC from the jejunum and colon suggest that the ICC from both organs share many similarities in membrane-related mechanisms.

### Construction of an ICC genome browser

We added the transcriptome data of ICC to our previously constructed the UCSC Smooth Muscle Genome Browser [16]. This genome browser, built in the UCSC Genome Browser [17], provides the genomic structure of each transcriptional variant, including promoter regions as well as each exon and intron for all genes expressed in ICC. This browser has also enabled us to analyze our transcriptome using genetic and epigenetic gene regulation data that have been compiled into the UCSC genome database by other investigators.

### Identification of ICC-specific genes

**Fig 3:**
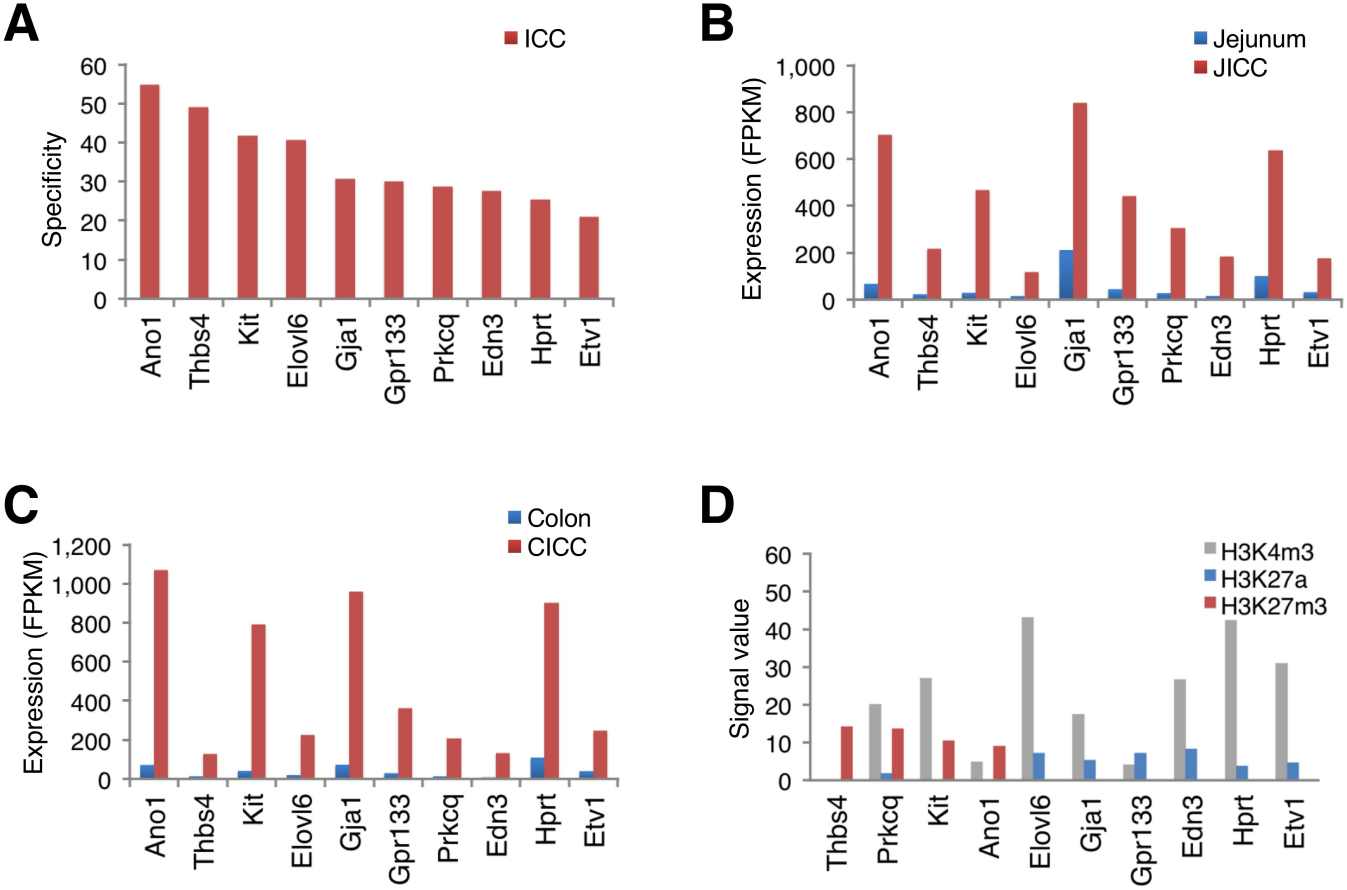
Identification of ICC-specific genes through expression profiles and histone modifications. (A) Specificity of ICC-enriched genes. Cell specificity was determined by comparative analysis of gene expression profiles among ICC, SMC, and PDGFRα^+^ cells: ICC^expression level (FPKM)^/[SMC^expression level (FPKM)^ + PDGFRα^+^ cells^expression level (FPKM)^]. (B and C) Comparison of JICC- and CICC-enriched gene expression. (D) Comparison of histone modifications on ICC-enriched genes. The signal value is the average of the mininum and maxium values of the ChIP-seq signals in the small intestine for each histone modification.

In order to identify the known and unknown distinctive cellular markers for ICC, genes preferentially expressed in ICC were analyzed. For use as a comparison, we have also obtained the transcriptome data from SMC [16] and PDGFRα^+^ cells (manuscript under review) from the murine jejunum and colon (Smooth Muscle Transcriptome Sequencing Project: http://med.unr.edu/physio/transcriptome). Comparative analysis of the expression profiles among ICC, SMC, and PDGFRα^+^ cells showed that ICC were indeed enriched in well-known ICC markers (*Ano1*, *Kit*, and *Prkcq*) as well as unknown, new genes (*Thbs4*, *Elovl6*, *Gja1*, *Gpr133*, *Edn3*, *Hprt*, and *Etv1*) (Fig 3A). All of these genes showed enriched expression, but the expression of *Ano1*, *Kit*, *Gja1*, and *Hprt* was more prevalent in both types of ICC when compared to corresponding tissue (Fig 3B and 3C). We further analyzed the ICC-enriched genes using the UCSC Smooth Muscle Genome Browser, which incorporated the small intestine histone modification maps (H3K4me3 for active or poised genes; H3K27a for active genes; and H3K27me3 for silenced genes) available at the public mouse encyclopedia of DNA elements (mouse ENCODE) [26]. We observed that SMC-specific genes had distinctive histone modification patterns when compared to ubiquitously expressed genes[16]. Likewise, the ICC-specific genes *Thbs4*, *Prkcq*, *Kit*, and *Ano1* have unique histone modification patterns (Fig 3D). Since the three genes *Prkcq*, *Kit*, and *Ano1* are already known markers of ICC, we further explored the new gene, *Thbs4*, enriched in ICC.

**Fig 4:**
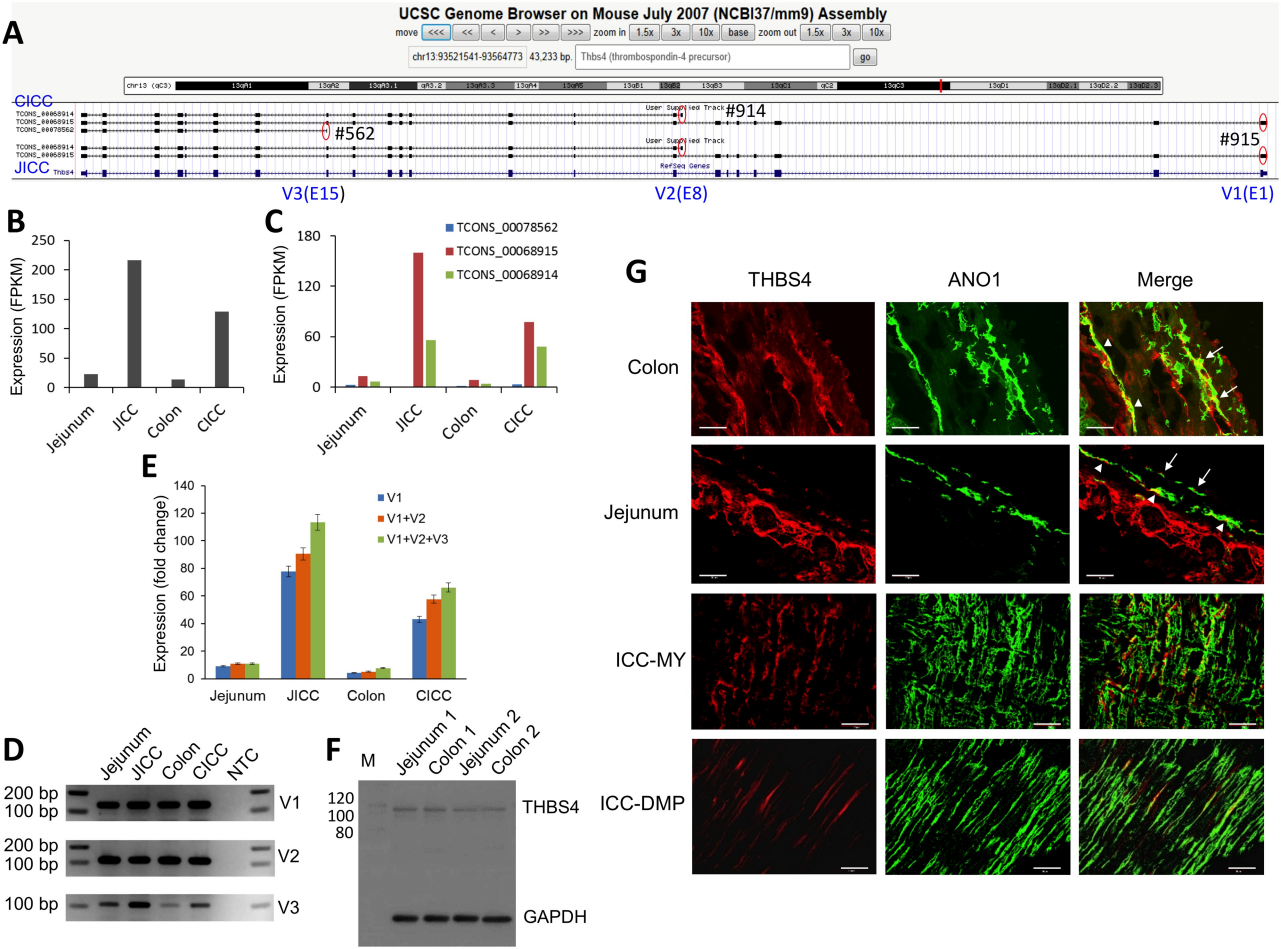
Identification of *Thbs4* as ICC-specific. (A) A genomic map view of three *Thbs4* variants (V1-3) expressed in JICC and CICC. Three alternative start exons (E1, E8 and E15) are circled (red). (B) Expression levels of total *Thbs4* mRNAs in JICC and CICC. (C) Expression levels of *Thbs4* transcriptional vaiants in JICC and CICC. (D) PCR validation of *Thbs4* exons with different transcriptional initiation sites in isolated ICC and in the muscularis of the murine jejunum and colon. NTC is non-template control. Primer sets were designed from variant exons in the regions of interest (see S11 Table for primer sequences). (E) Expression levels of V1, V1+V2, and V1+V2+V3 mRNAs in isolated ICC and muscularis of the murine jejunum and colon were measured by qPCR (see S11 Table for primer sequences). Expression levels of *Thsb4* variants were normalized using the endogenous control, *Gapdh*. (F) Western blot analysis using an N-terminal THBS4 antibody, showing THBS4 protein expressed in the muscularis of the murine jejunum and colon (1, 1 month and 2, 2 months). (G) Detection of THBS4 protein in colonic and jejunal ANO1^+^ ICC-MY, ICC-SMP, and ICC-DMP. Cryosection images (top 2 panels) showing THBS4 was detected in ANO1^+^ ICC-MY (arrows in colon and jejunum), ICC-SMP (arrow heads in colon) and ICC-DMP (arrow heads in jejunum). Whole mount images (bottom 2 panels) also showing the protein was found in ANO1^+^ ICC-MY and ICC-DMP. All scale bars are 50 μm.

*Thbs4* encodes the thrombospondin family member 4 of extra cellular adhesive glycoproteins that mediate cell-to-cell and cell-to-matrix interactions [27]. Three variants of this gene were expressed and alternatively started on different exons: E1 (V1, TCONS_00068915), cryptic E8 (V2, TCONS_00068914), and E15 (V3, TCONS_00078562) (Fig 4A). Expression of *Thbs4* was highly enriched in JICC and CICC (Fig 4B). Expression levels of the variants were V1>V2>V3 (Fig 4C), of which V1 is the longest (3,202 bp) (S2 Table) encoding 22 exons. Expression of all three variants of the gene were validated in ICC of both organs by RT-PCR (Fig 4D) and qPCR (Fig 4E). To access the primary structure of protein of the three *Thbs4* variants, the predicted peptide sequence of each THBS4 variant was obtained from their prospective open reading frame. The amino acid sequences of all THBS4 variants (V1, V2, & V3) were aligned with each other in order to identify conserved domains and regions (S2 Fig). The only full-length THBS4 peptide is V1 (963 aa) which contains an N-terminal signal peptide, as well as a Lamin G-like domain, two cell attachment site motifs, four EGF-like domains (EGF-like 1–4: EGF-like 3 and 4 contain calcium binding site), eight repeat domains also found in the thrombospondin type 3 and the C-terminus containing a domain (TSP C-terminal) that is highly conserved among the thrombospondin family. The other two variants TCONS_00068914 (V2, 601 aa) and TCONS_00078562 (V3, 205 aa) are truncated at the N-terminus. The V2 begins at the EGF-like 4 domain and continues to the end of the ORF while the V3 only contains a part of the TSP-terminal region (S2 Fig). A protein with a molecular mass of ~106 KDa was detected by Western blot targeting THBS4, suggesting that the protein was translated from V1 as the predicted molecular mass of V1 is 106.39 (66.35 kDa for V2, and 23.68 kDa for V3) (Fig 4F) [28]. Finally, in order to show THBS4 localization, colonic and jejunal and smooth muscle tissue was immunohistochemically probed with ANO1 and THBS4 antibodies. THBS4 protein was detected in ANO1^+^ ICC-MY and ICC-SMP (ICC within the submucosal plexus) in the colon and ANO1^+^ ICC-MY and ICC-DMP in the jejunum (Fig 4G). THBS4 protein was expressed in ICC but at a lower level than ANO1 was expressed. This finding was corroborated by the levels of their respective mRNAs as shown in S3 Table. The protein was abundantly expressed in ICC-MY and ICC-DMP as early as embryonic day E14, but its expression gradually decreased from birth into adulthood (unpublished data), suggesting that THBS4 may be required for ICC growth but not once the cells are fully differentiated. In addition, THBS4 was detected within the cytoplasm of some ICC as well as in the extracellular regions around the ICC, suggesting that THBS4 was secreted and localized to regions around the cells. Furthermore, THBS4 was detected in serosal mesothelial cells, other muscle interstitial cells, and submucosal cells which were ANO1 negative.

### Comparative analysis of ion channels and transporters

**Fig 5:**
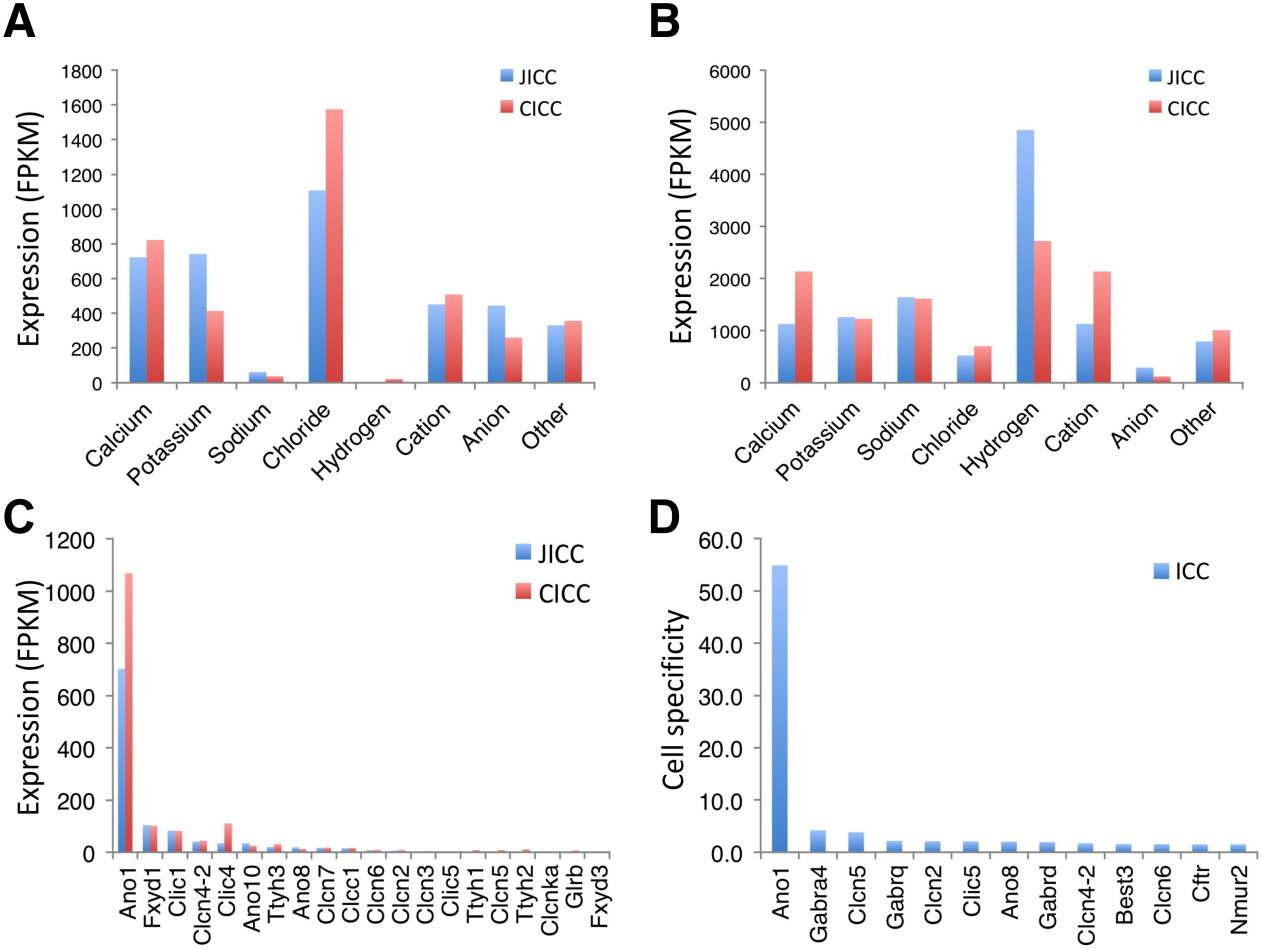
Comparison of ion channel and transporter isoform genes expressed in ICC. (A) Comparison of expression levels of ion channel isoforms in JICC and CICC. (B) Comparison of expression levels of ion transproter isoforms in JICC and CICC. (C) Chloride channel isoforms enriched in JICC and CICC. (D) ICC-specific chloride channel isoforms. Cell specificity was determined by comparative analysis of gene expression profiles among ICC, SMC, and PDGFRα^+^ cells.

ICC are the pacemaker cells that generate electrical slow waves in the GI tract [29–31], thus, it has been of interest to identify the ion channels and transporters responsible for pacemaker activity and the ability of ICC to actively propagate slow waves. We identified a total of 550 ion channel isoforms and 527 ion transporter isoforms in isolated ICC. The full list of ion channel and transporter isoforms can be found in S4 Table. JICC expressed 317 ion channels, 210 ion transporters, and 23 coupled ion channels/transporters. CICC expressed similar numbers of ion channel and transporter isoforms, yet a bit less than JICC: 308 ion channels, 198 ion transporters, and 21 coupled ion channels/transporters. Interestingly, Cl^-^ channels were the most highly expressed type of ion channel in both JICC and CICC, followed by Ca^2+^ channels (Fig 5A). It is interesting to note that H^+^ transporters were the most highly expressed transporters in ICC of both organs (Fig 5B). To identify the most abundantly expressed isoforms of ion channel and transporter classes in ICC, a more detailed analysis was performed.

### Ion channels: Chloride channels

**Fig 6:**
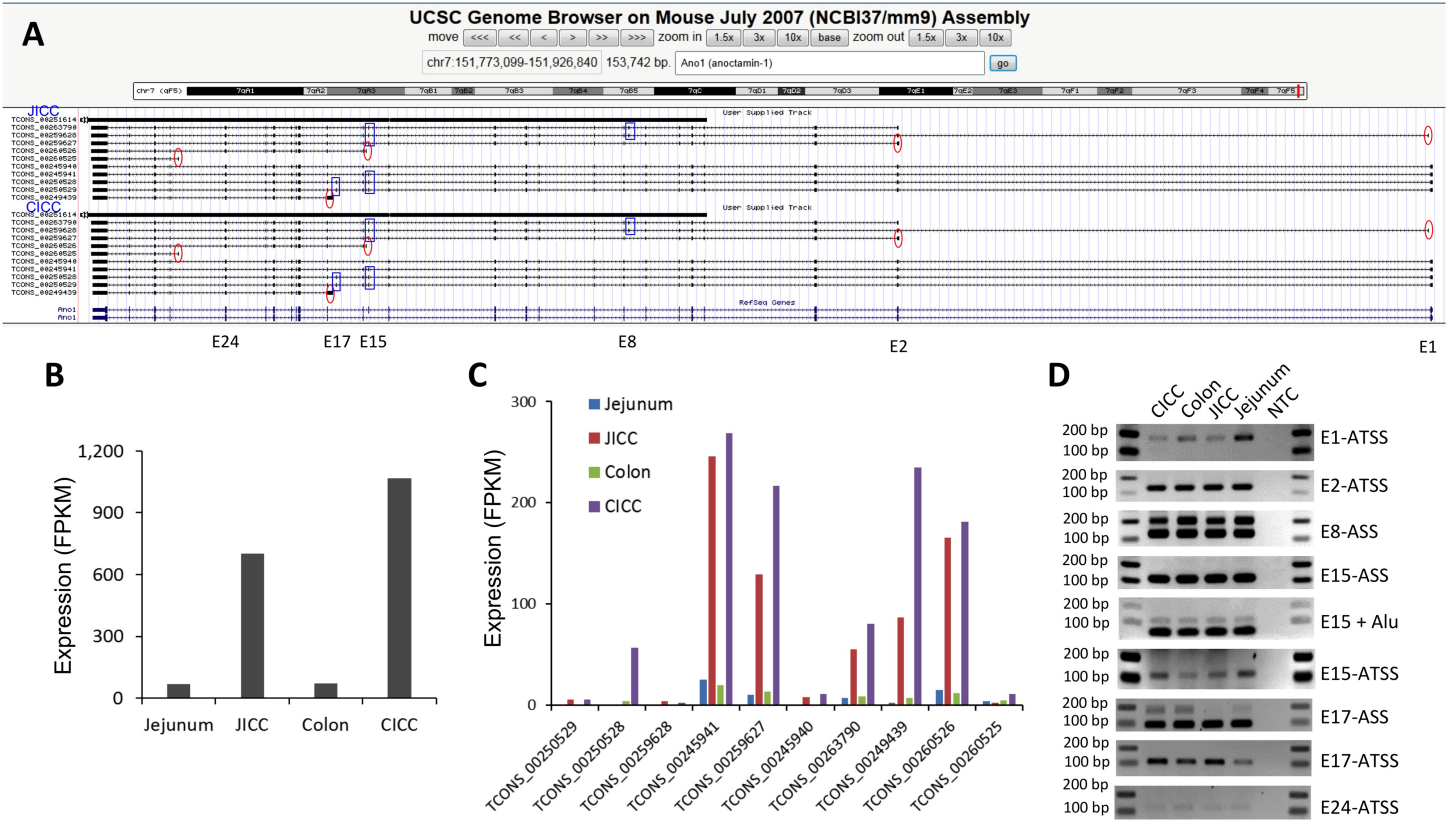
Identification of multiple *Ano1* transcriptional variants. (A) A genomic map view of 10 *Ano1* variants expressed in JICC and CICC. Five alternatively starting exons (E1, E2, E15, E17, and E24) are circled and three differentially spliced exons (E8, E15, and E17) are boxed. (B) Expression levels of total *Ano1* mRNAs in JICC, CICC and tissues of origin. (C) Expression levels of *Ano1* transcriptional variants in JICC, CICC and tissue of origin. (D) PCR validation of *Ano1* exons with alternative transcriptional start sites (ATSS) and alternative spliced sites (ASS) in isolated ICC and their corresponding tissue. E8-ASS, E15-ASS, and E17-ASS contained 2 bands amplified from spliced variants. PCR products of E15-ASS were digested by Alu to distinguish E15 skipping transcripts (undigested) from E15 containing transcripts (digested). NTC is non-template control. Primer sets were designed from variant exons in the regions of interest (see S11 Table for primer sequences).

Both JICC and CICC expressed *Ano1* abundantly and specifically (Fig 5C and 5D). *Ano1* (aka *Tmem16a*) encodes a Ca^2+^-activated Cl^-^ channel that is fundamental to pacemaker activity in GI muscles [32–35]. However, other Cl^-^ channel subunits such as *Fxyd1*, *Clic1*, *Clcn4-2* and *Clic4* were also expressed in ICC, but at much lower levels than *Ano1*. Analysis *of Ano1* within the UCSC Smooth Muscle Genome Browser showed that *Ano1* was transcribed into as many as ten different transcriptional variants in ICC of both organs (Fig 6A). There are five different cryptic/partial exons (after E1, E2, E15, E17, and E24) that begin each isoform, making each variant truncated at the 5’ end besides those that start with E1. There are also three exons E8, E15, and E17 that are alternatively spliced depending on isoform type. mRNA expression levels of *Ano1* in JICC and CICC were much higher than observed in corresponding tissues (Fig 6B). The dominant variant was TCONS_00245941 (4,601 bp) being one of the longest variants that starts with exon 1 (Fig 6C and S2 Table). Other variants TCONS_00259627 (E2 variant), TCONS_00263790 (E2 variant), TCONS_00249439 (E17 variant), and TCONS_00260526 (E15 variant) were also highly expressed in both ICC groups. Interestingly, TCONS_00250528 (E1 variant) was expressed in CICC, but not JICC. The alternatively initiated and differentially spliced exons were validated using variable exon-specific primer sets with RT-PCR techniques on mRNA isolated from JICC, CICC and their corresponding tissue (Fig 6D). All PCR amplicons, from alternative transcriptional start and splice sites, matched the length of expected exon variants.

**Fig 7:**
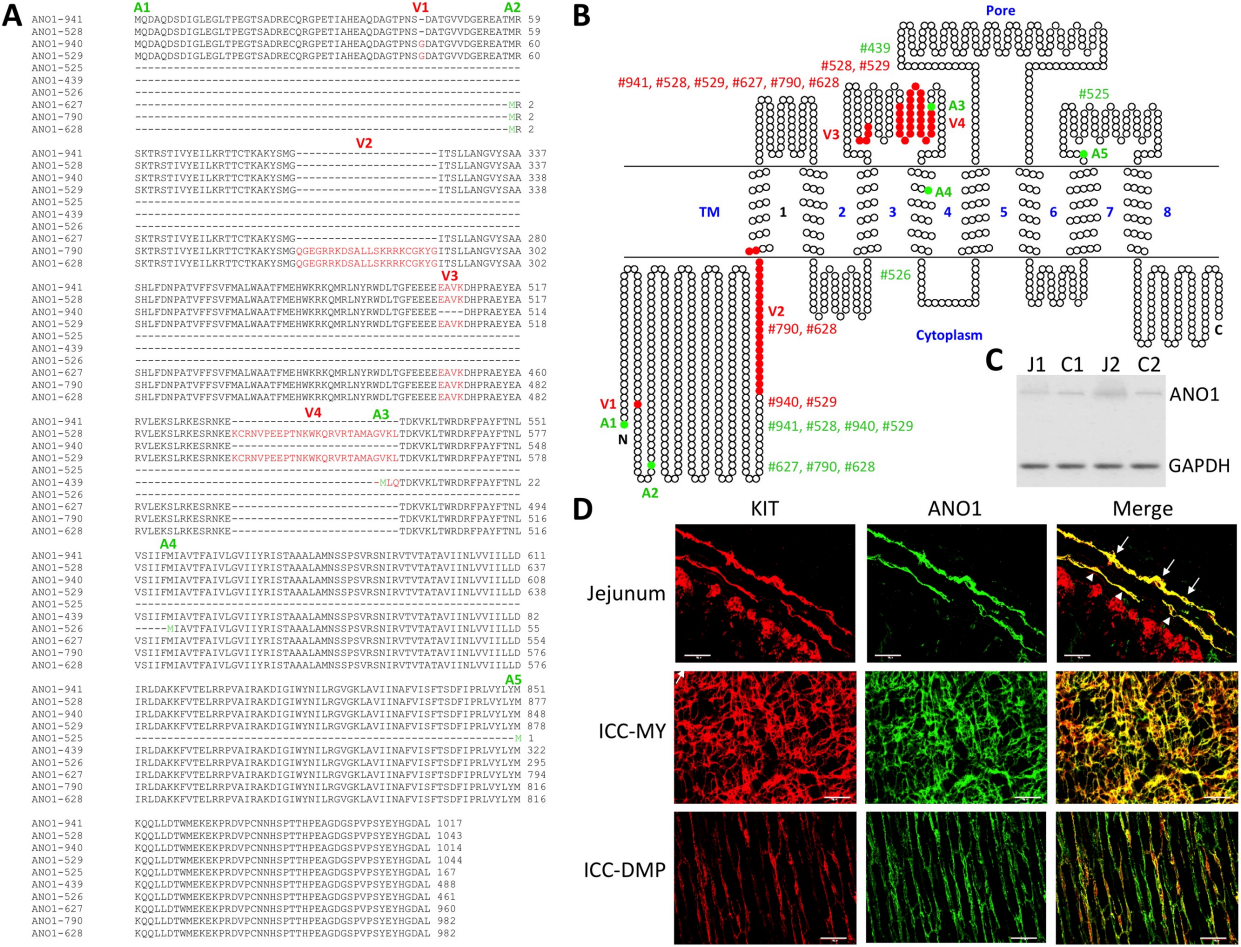
Identification of ANO1 protein. (A) Alignment of predicted amino acid sequences of ANO1 transcriptional variants. The open reading frame was identified for each transcriptional variant, and predicted amino acid sequences were aligned. Alternatively starting methionines (M) are indicated by a green color and differentially spliced residues by a red color. Last three numbers from variant IDs of RNA-seq data were used (e.g. ANO1-941, TCONS_00245941). (B) A topological map of ANO1 variants. Each circle denotes the corresponding amino acid. Colors on amino acid sequence show distinct regions and domains: red, missing or inserted peptides from differentially spliced exons; green, start codons found in differentially spliced variants. Alternatively starting methionines, insertion, or deletion in each ANO1 variant are indicated with variant IDs. Eight transmembrane domains 1–8 and a pore region are shown. (C) Western blot analysis showing ANO1 protein expressed in jejunum (J) and colon (C) muscularis (1, 1 month and 2, 2 months). (D) Detection of ANO1 protein in jejunal KIT^+^ ICC-MY and ICC-DMP. Cryosection images (top panels) showing ANO1 detected in KIT^+^ ICC-MY (arrows) and ICC-DMP (arrow heads). Whole mount images also showing the protein found in KIT^+^ ICC-MY (middle panels) and ICC-DMP (bottom panels). All scale bars are 50 μm.

Amino acid (aa) sequences of all ten *Ano1* transcriptional variants were further analyzed by aligning all putative variant protein sequences. The variants encode polypeptides ranging from 167 to 1044 aa in length. There are five groups of variant polypeptides with alternative start codons (A1-5) and four regions (V1-4) of variable peptides generated from alternatively spliced exons (Fig 7A). The full-length transcript group starting from exon 1 includes TCONS_00245941 (ANO1-941), ANO1-528, ANO1-940, and ANO1-529. Among these transcripts, ANO1-529 is the longest variant coding for a polypeptide that is 1044 aa in length and contains three of four variable regions (V1,V3, and V4) (Fig 7A). ANO1-529 consists of a long cytoplasmic N-terminus, a short cytoplasmic C-terminus, and eight transmembrane domains, including a pore region (Fig 7B). The channel topology of the ANO1 protein shows the gain or loss of several amino acid sequences based on which isoform is being translated (Fig 7B). These regions of differential expression appear to be in either the N-terminus, or an extracellular loop between transmembrane 3 and 4 (Fig 7B). An ANO1 protein, with a molecular weight of ~114 kDa was detected by Western blot, suggesting that the protein was most likely translated from E1 (ANO-941) and/or E2 variants (ANO1-790 and ANO1-627) based on expression levels in both ICC (Fig 6C) as well as predicted molecular weight (Fig 7A and 7C): 116.81 kDa for ANO-941, 113.41 kDa for ANO1-790, and 110.93 kDa for ANO1-627 [28]. Finally, localization of ANO1 was examined in jejunal smooth muscle using immunohistochemistry. The protein was found to co-localize with KIT^+^ ICC-MY and ICC-DMP (Fig 7D), as previously reported [36].

### Ion channels: Calcium, potassium, cation, and sodium channels

ICC expressed two types of voltage-dependent calcium channels: *Cacna1h* (T-type) and *Cacna1d* (L-type). Interestingly, *Cacna1h* was predominantly expressed in JICC while *Cacna1d* was predominantly expressed in CICC (S3A Fig). Additionally, an intracellular IP3-gated calcium channel (*Itpr1*) and an endoplasmic reticulum calcium sensor (*Stim2*) were both found to be expressed at a higher rate in CICC than in JICC. Other calcium channels and calcium sensing regulators such as voltage-dependent calcium channels (*Cacng7*, *Cacnb3*), a polycystin cation channel (*Pkd1*), a regulator of the store-operated calcium channel Saraf (*Tmem66*), and a calcium sensing protein (*Gas6*) were found to be highly upregulated in both JICC and CICC (S3A Fig). Interestingly, all of the voltage-dependent calcium subunits (*Cacng6*, *Cacng8*, *Cacng7*, *Cacng4*, *Cacng3*, and *Cacng2*) were expressed in an ICC-specific manner (S3B Fig). Among the calcium subunits, *Cacng7* was most abundantly expressed in both JICC and CICC (S3A Fig). CACNG7 is a neuronal voltage-dependent calcium channel γ subunit that regulates the trafficking and gating properties of AMPA-selective glutamate receptors [37, 38]. Voltage-dependent calcium channels are formed as a complex of several subunits including the γ subunit [39]. Neural L-type channels contain the γ2 subunit [40]. CACNG7 is possibly a subunit that may bind to the neural form of L-type calcium channel CACNA1D in ICC.

ICC expressed as many as 93 potassium channel subunits (S4 Table). Acid-sensitive potassium channels (*Kcnk3*) and rapidly activating-delayed rectifier potassium channels (*Kcnh2*, known as ERG) were predominantly expressed in both groups of ICC (S3C Fig). ICC-specific potassium channel subunit genes included voltage-gated subfamily G potassium channel (*Kcng3*), ATP-sensitive potassium channel subunit (*Abcc8*), inward-rectifier potassium channel (*Kcnj2*), and large conductance calcium-activated potassium channel (known as BK) β subunit (*Kcnmb2*) (S3D Fig). Interestingly, the ICC-predominant and ICC–specific potassium channel subunit genes were expressed more in JICC than in CICC.

**Fig 8:**
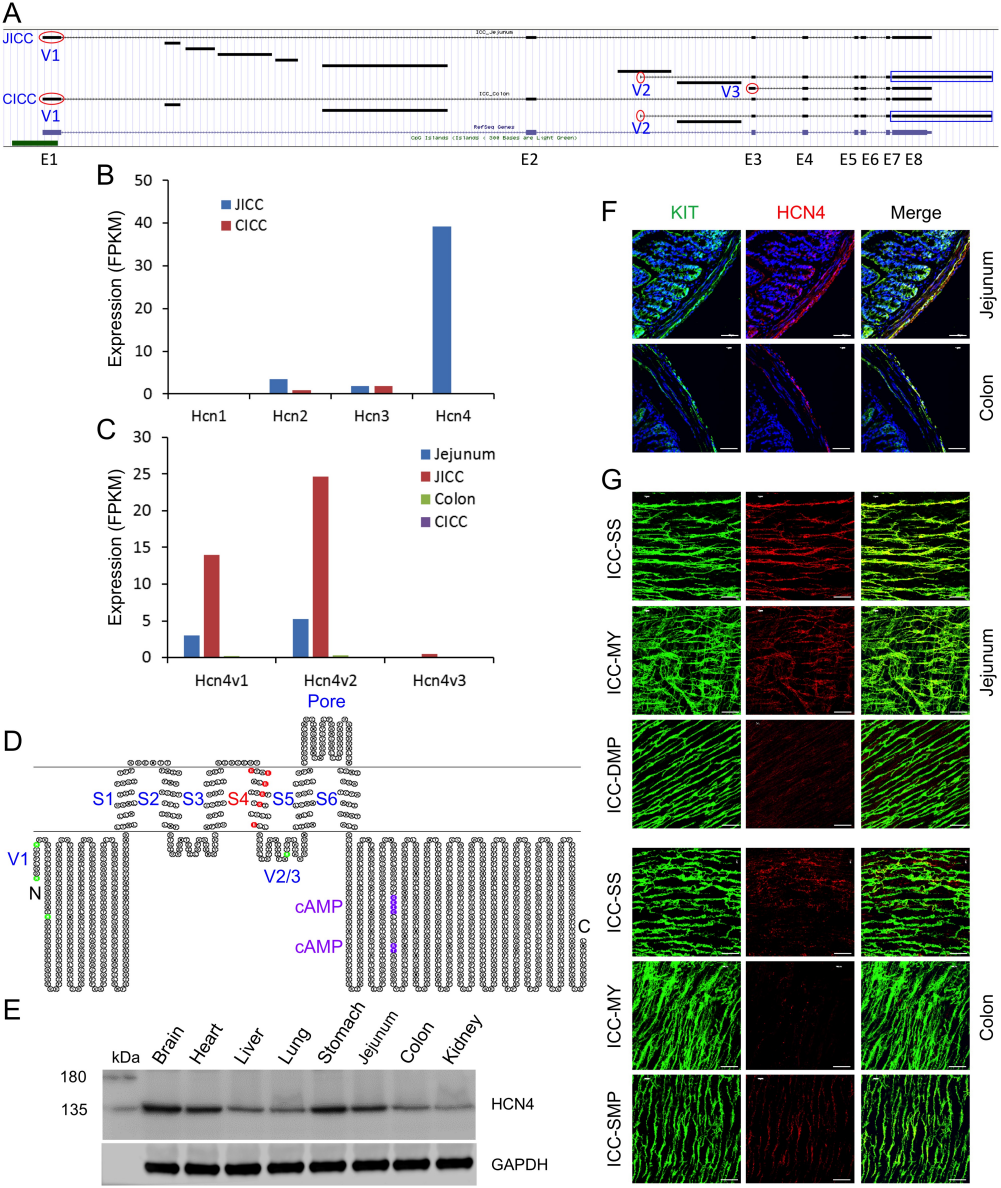
Identification of ICC-specific *Hcn4*. (A) A genomic map view of *Hcn4* variants expressed in JICC and CICC. Three alternative initial exons (V1-3) are circled in red and the differential last exon (E8) is boxed in blue. (B) Expression levels of total *Hcn* isoform genes in JICC and CICC. (C) Expression levels of *Hcn4* transcriptional vaiants in JICC and CICC. (D) A topological map of HCN4 variants. Each circle denotes a single amino acid. Colors on amino acid sequence show distinct regions and domains. Green represents start codons found in alternatively initiated variants (V1-3). Six transmembrane domains (S1-6) and a pore region are shown. Red represents voltage sensor residues in S4. Two cAMP binding sites are in purple. (E) Western blot analysis showing HCN4 protein expressed in multiple tissues including stomach, jejunum, and colon muscularis. (F) Cryosection images of HCN4 protein in jejunum and colon. HCN4 was detected in the KIT^+^ serosal layer, myenteric plexus, and deep muscular plexus in jejunum. It was also detected in the serosal layer and submuscular plexus in colon with much lower levels. (G) Whole mount images of HCN4 protein in jejunum (top panels) and colon (bottom panels) showing the protein found in KIT^+^ ICC-SS, ICC-MY, and ICC-DMP in jejunum, and KIT^+^ ICC-SS AND ICC-SMP in colon. All scale bars are 50 μm.

Expression levels of sodium channels in ICC were generally lower than those of other ion channels (Fig 5A). The most predominant sodium channel gene was the voltage-gated type I sodium channel β subunit (*Scn1b*) (S3E Fig). Other ICC–specific sodium channel genes included voltage-gated type IV sodium channel α subunit (*Scn4a*) and voltage-gated type I sodium channel α subunit (*Scn1a*) (S3F Fig). Cation channel subunits enriched in JICC and CICC included polycystic kidney disease 2 (*Pkd2*), solute carrier family 8 (sodium/calcium exchanger) member 1 (*Slc8a1*), and cholinergic receptor nicotinic β polypeptide 1 (*Chrnb1*), all of which were differentially expressed between the cell groups. *Pkd2* and hyperpolarization-activated cyclic nucleotide-gated K^+^ channel 4 (*Hcn4*) were predominantly expressed in JICC while *Slc8a1* and *Chrnb1* were expressed much higher in CICC (S3G Fig). ICC–specific cation channel subunits were: *Hcn4*, transient receptor potential cation channel subfamily M member 5 (*Trpm5*), glutamate receptor ionotropic N-Methyl D-Aspartate 2A (*Grin2a*), and transient receptor potential cation channel subfamily V member 1 (*Trpv1*) (S3H Fig). *Hcn4* was of particular interest since it is known as a “pacemaker channel” expressed in the heart and central nervous system [41, 42]. There are four *Hcn* isoform genes, among which, three isoforms *Hcn2*, *Hcn3*, and *Hcn4* were expressed in ICC (S4 Table). *Hcn4* was the predominantly expressed isoform in JICC, but expressed at very low levels in CICC (S4 Table and Fig 8B). This gene, encoding eight exons, was transcribed into three different variants, *Hcn4v1*, *Hcn4v2*, and *Hcn4v3*, alternatively starting at exon 1, intron 2, and intron2/exon3, respectively (Fig 8A). *Hcn4v1* is the same as the known reference gene, but *Hcn4v2* and *Hcn4v3* are new variants found in JICC. The expression levels were *Hcn4v2*>*Hcn4v1* in JICC and *Hcn4v3* had very little expression (Fig 8C). Amino acid (aa) sequences of all thee *Hcn4* transcriptional variants were further analyzed by aligning all putative variant protein sequences. The variants encode two polypeptides (a full length 1201 aa and a truncated 795 aa); HCNV1 encodes up to 1201 aa, and HCN4V2 and HCNV3 encode 795 aa (S4 Fig). The start codon (M) of HCNV1 is not clear since M starts at the end of exon1 without any 3’ UTR sequence. There are three putative start codons at the N-terminus in the full length peptide (S4 Fig). The full length peptide consists a cytoplasmic N-terminus, 6 transmembrane domains (S1-6) and a long cytoplasmic C-terminus (Fig 8D). The S4 transmembrane domain includes 6 positively charged residues, used as voltage sensors, suggestive of the voltage-dependency of this channel. In addition, the C-terminus has two putative cyclic nucleotide cAMP binding sites, suggestive of dependency on cAMP for channel gating. The truncated peptide encoded by *Hcn4v2* and *Hcn4v3* consists of the transmembrane domains S5 and S6, the pore region, and the long N-terminus (Fig 8D). Interestingly, *Hcn4v2*, which encodes the truncated peptide, was the dominant variant expressed in JICC (Fig 8C). Protein expression was examined in multiple tissues including jejunum and colon by Western blot. A single bend of HCN4 protein, with a molecular weight of ~135 kDa, was detected in multiple tissues including brain, heart, liver, lung, stomach, jejunum, colon, and kidney (Fig 8E), suggesting that the protein was most likely translated from HCNV1 (full-length variant) (Fig 8A). Expression of the protein in brain, heart, stomach, and jejunum was more highly detected than in the other listed tissues. In addition, HCN4 protein expression being higher in jejunum than colon was in agreement with mRNA expression (Fig 8B). The cross section images showed that HCN4 protein was localized within KIT^+^ cells of the serosal layer, longitudinal muscle layer, myenteric plexus, deep muscular plexus, and submuscular plexus in jejunum and colon (Fig 8F). The whole mount images confirmed that the protein was expressed in ICC-SS, ICC-MY (Fig 8G). It was also detected within the same cell types (ICC- ICC-SS, ICC-MY, and ICC-SMP) in the colon, but it was at much lower levels.

### Transporters: Hydrogen and sodium transporters

Hydrogen transporter isoforms were the main class of transporters expressed in JICC and CICC (Fig 5B). The dominant isoforms expressed in both ICC are H^+^ transporting ATP synthase subunits that consist of mitochondrial F1 and F0 complexes (S5A Fig). Among them, H^+^ ATP synthase mitochondrial F1 complex β (*Atp5b*) was the most abundantly expressed in both JICC and CICC. Interestingly, JICC and CICC specifically expressed gastric H^+^/K^+^ ATPase α (*Atp4a*) and cation proton antiporter 2 (*Nhedc2*) (S5B Fig). The dominant isoforms of sodium transporter isoforms included Na^+^/K^+^ ATPase α1 (*Atp1a1*), Na^+^/K^+^/Cl^-^ symporter (*Slc12a2*), Na^+^/K^+^ ATPase α2 (*Atp1a2*), and Na^+^/K^+^/Ca^2+^ exchanger (*Slc24a3*) (S5C Fig). Both JICC and CICC specifically expressed *Slc4a4* (Na^+^/HCO3^-^ cotransporter) (S5D Fig).

### Comparative analysis of growth factors, receptors, and transcription factors

THBS4 has growth factor activity in that it stimulates cell growth and proliferation of erythroid cells [43] and recognizes integrin α_M_β_2_ as its receptor [44]. Using our transcriptome, we searched for growth factors that were expressed in ICC. A total of 52 growth factors were expressed in JICC and CICC (S5 Table). The genes associated with growth factor activity, which also had high levels of expression in ICC, included glucose phosphate isomerase 1 (*Gpi1*) and *Thbs4* (S6A Fig). *Thbs4* was the most ICC specific growth factor gene (S6B Fig). Bone morphogenetic protein 7 (*Bmp7*) and fibroblast growth factor 12 (*Fgf12*) were also expressed in an ICC-specific manner. Next, we analyzed what types of receptors were expressed in ICC. A total of 66 receptors were expressed in JICC and CICC (S6 Table). Stem cell factor receptor (*Kit*) and fibroblast growth factor receptor 1 (*Fgfr1*) were among the most highly expressed receptor genes (S6C Fig). As expected, *Kit* was the most specific to JICC and CICC, followed by Eph receptor A7 (*Epha7*) (S6D Fig). Next, we elucidated the transcription factors expressed in ICC. A total number of 137 transcription factors were identified from ICC (S7 Table). FBJ murine osteosarcoma viral oncogene homolog (*Fos*) and catenin (cadherin-associated protein) β1 (*Ctnnb1*) were the most highly expressed transcription factors in both JICC and CICC (S6E Fig). ICC-specific genes included zinc finger homeobox 3 (*Zfhx3*), LIM domain only 2 (*Lmo2*), and forkhead box M1 (*Foxm1*) (S6F Fig). These ICC-specific transcription factors may play a role in driving cell-restricted gene expression.

### Comparative analysis of epigenetic enzymes and regulators

Gene expression is profoundly regulated by the activity of epigenetic enzymes [45]. One epigenetic mechanism involved in regulating gene expression is the methylation and demethylation of DNA [45]. ICC expressed two DNA methyltransferases (*Dnmt1*, *Dnmt3a*), all three Tet methylcytosine dioxygenases (*Tet1*-*3*), and a DNA oxidative demethylase (*Alkbh1*) (S8 Table). *Dnmt3a* and *Tet2* were the dominant isoforms expressed in CICC and JICC, respectively (S7A Fig) while *Tet2*, *Tet1* and *Dnmt3a* were slightly ICC-specific (S7B Fig). We also searched methyl-CpG-binding domain (MBD) proteins that bind to DNA containing methylated cytosines at CpG sites. The predominant MBD protein genes expressed in ICC include *Mbd3*, *Mbd2*, and *Mbd6* (S7C Fig). The transcripts of *Mbd6* and E3 ubiquitin-protein ligase (*Uhrf1*) were found to be ICC-specific (S7D Fig). Other biochemical pathways involving the epigenetic regulation of gene expression are the acetylation/deacetylation and methylation/demethylation of histone proteins [46]. We identified 39 genes expressed in ICC associated with histone acetyltransferase activity (S8 Table). Among them, O-linked N-acetylglucosamine (GlcNAc) transferase (*Ogt*) was highly, and restrictively, expressed in both JICC and CICC (S8A and S8B Fig). There were 16 genes expressed in ICC that are associated with histone deacetylases (S8 Table). The predominant histone deacetylases in ICC included *Hdac5* and *Hdac1* (S8C Fig) while *Hdac4* and *Hdac1* appeared to be ICC-specific (S8D Fig). We also identified 24 genes expressed in ICC that are associated with histone methyltransferase activity (S8 Table). Among them, suppressor of variegation 4–20 homolog 2 (*Suv420h2*) was highly, and selectively, expressed in both JICC and CICC (S8E and S8F Fig). Lastly, we found 15 genes that are associated with histone demethylase activity (S8 Table). We found *Kdm1a* to be the most highly expressed in both groups of ICC (S8G Fig), and jumonji AT rich interactive domain 2 (*Jarid2*) was the most ICC-specific (S8H Fig).

### Comparative analysis of protein kinases and phosphatases

Protein activity can be regulated by adding or removing phosphate groups to specifically targeted proteins [47]. We searched genes associated with protein kinase and/or phosphatase activity. A total of 354 protein kinase genes were expressed in ICC (S9 Table). The predominant kinase genes found in ICC included *Kit*, myosin light polypeptide kinase (*Mylk*), protein kinase Cθ (*Prkcq*), and dystrophia myotonica-protein kinase (*Dmpk*) (S9A Fig). *Kit*, *Prkcq*, obscurin cytoskeletal calmodulin and titin-interacting RhoGEF (*Obscn*), and bone morphogenetic protein receptor type 1B (*Bmpr1b*) were shown to be the most specific to ICC (S9B Fig). We also found a total of 105 protein phosphatase genes in ICC (S10 Table). Among them protein phosphatase 1, catalytic subunit β (*Ppp1cb*) and γ (*Ppp1cc*) were the most highly expressed in ICC (S9C Fig). ICC-specific phosphatase genes included pyruvate dehyrogenase phosphatase catalytic subunit 2 (*Pdp2*), CDC14 cell division cycle 14B (*Cdc14b*), and dual specificity phosphatase 4 (*Dusp4*) (S9D Fig).

## Discussion

ICC serve as electrical pacemakers and generate electric slow waves in GI smooth muscles. ICC predominantly express KIT which is required for several cellular functions and survival. Identification of ICC with anti-KIT antibodies has greatly advanced studies in GI physiology and pathology. However, immunohistochemical techniques limited researchers to only morphological studies of the cells. We overcame this limitation by generating *Kit*^*+/copGFP*^ mice that label all subtypes of ICC with copGFP [13]. Expression of the reporter is a powerful tool that can be used to study cellular changes involved in the development of GI diseases in animal models, as well as characterizing the electrophysiological properties of ICC. In the present study we utilized FACS to purify ICC from the murine jejunum and colon, and this allowed quantitative examination of gene expression by RNA-Seq and qPCR. We obtained full transcriptomes of ICC populations in these organs, and this has provided unprecedented information regarding the genetic identity of these cells.

ICC expressed as many as 18,045 genes which accounts for ~75% of the entire murine genome (Fig 2A). Highly expressed genes included those associated with many GO categories involved in membrane regulation/function, suggesting the importance of membrane functions in these excitable cells (Fig 2C). JICC share 91% (16,500) similarity in gene expression with CICC and the expression profiles between the two groups are very similar (r = 0.88). The same subpopulation of ICC-MY is found in JICC and CICC, but intramuscular ICC (ICC-DMP in JICC and ICC-IM in CICC) and submucosal ICC (ICC-SMP in CICC) are found in different anatomical locations and their morphologies are also different. These different subtypes may account for some of the differential gene expression in these organs (i.e. 1,545 genes in JICC and 403 genes in CICC in Fig 2A).

We previously reported the jejunal and colonic murine SMC transcriptomes and used that data to build the interactive SMC genome browser [16]. The JICC and CICC transcriptomes were an addition to the sequencing project of SMC, ICC, and PDGFRα^+^ cells, called SIP cells. The SIP cells are electrically coupled via gap junctions and form an electrical syncytium [48]. The transcriptomes of SIP cells were similar (sharing 14,845 genes in jejunal SIP and 14,483 in colonic SIP), suggesting that they have interdependent cellular function and process. Yet, there are several hundred genes that were restrictively expressed in each cell type of SIP cells. These restricted genes are involved in a unique function of each type of SIP cell.

ICC expressed a large number of ion channel and transporter genes (550 ion channel and 527 transporter isoforms), adding support to the hypothesis that the function of ICC is rooted in electrical pacemaker activity and neurotransmission. Although the number of expressed ion channel genes were similar in JICC and CICC, overall expression patterns of the genes were less similar (r = 0.84) than the entire transcriptome (r = 0.88). In fact, expression levels for some ion channel isoform were quite different (S4 Table). For example, *Cacna1h* was predominantly expressed in JICC (FPKM of 110 in JICC and only 7 in CICC). This difference may partially explain some of the differences in electrical behaviors between the jejunum and colon. JICC generate electrical slow waves with 60 mV upstroke depolarizations [49, 50], whereas slow waves in CICC are likely to be much smaller in amplitude. Electrical recordings from direct impalements of ICC in colonic muscles have not been reported, but slow waves recorded in SMC are only a few mV in amplitude [51]. *Chrnb1* was expressed dominantly in CICC (9 in JICC and 105 in CICC). This gene encodes a subunit of nicotinic receptors, but the role for such receptors and whether intact channels form in ICC is unknown. *Ano1* was the most highly, and specifically, expressed channel gene in ICC. Previous studies have shown that *Ano1* encodes for a Ca^2+^-activated Cl^-^ channel that is fundamental for pacemaker activity and generation of spontaneous transient inward currents in the intestine [15, 52]. ICC expressed as many as 10 transcriptional variants of *Ano1* (Fig 6A), which were generated by differential transcription start sites, alternative splicing, or deletion/insertion events in transcript formation that may alter subsequent protein expression (Fig 7A). Even though ANO1 was detected in all ICC, specialized ICC subtypes have particular functions. For example, ICC-MY in the small intestine are involved in slow wave generation [2, 5] while the role of ICC-DMP is mediation of inputs from enteric motor neurons [53]. As observed in the transcript data, many additional ion channel genes are expressed, but electrical recordings from freshly isolated ICC have failed to record currents indicative of the conductance levels encoded by these genes (e.g. *Kcnj2*), suggesting either the need for further electrophysiological studies or that many transcripts fail to be translated into functional membrane proteins. It is also interesting to find prominent expression of channel subunits (e.g. *Cacng6*, *Cacng8*, *Cacnb4*, *Kcng3*, *Abcc8*, and *Kcnmb2*) expressed without robust expression of pore forming α or other subunits. The reason for expression of these genes is also currently unknown. HCN channels participate in pacemaker activity in cardiac myocytes [54] and neurons [55]. *Hcn4* was the dominant isoform found in ICC (Fig 8B). Mutation of HCN4 in humans is associated with cardiac arrhythmias [56] and the inducible, and cardiac-specific, *Hcn4* knockout in mice leads to severe bradycardia, cardiac arrest, and death [57]. HCN channel currents were priviously reported in studies of ICC from the murine gastric antrum [58] and colon [59], but no studies have observed this conductance in ICC of the small intestine. The protein was detected in the stomach, jejunum and colon (Fig 8E). Its expression in stomach was even higher than in heart. Consistent with the transcriptome data of JICC and CICC, the protein was detected more in the jejunum than in the colon. We confirmed HCN4 was specifically expressed in ICC-SS, ICC-MY, and ICC-DMP in jejunum or ICC-SMP in colon (Fig 8G). ICC-MY and ICC-IM produce slow waves in the stomach and small intestine, whereas slow waves in the colon are largely produce from ICC-SMP [60]. It is of great interest to see if HCN4 channel contributes to the electrical rhythmicity or if it plays role in regulating the pacemaker activity in the GI tract. Further investigations will be necessary to address this question.

Another ICC-restricted gene identified in this study was *Prkcq*. *Prkcq* encodes protein kinase C theta (PKCθ), and the protein product of this gene was found to be expressed exclusively in ICC-MY and ICC-IM of guinea-pigs [61]. We confirmed that *Prkcq* was highly and selectively expressed in murine JICC and CICC. PKCθ protein was predominantly detected in guinea-pig ICC-MY which are involved in pacemaker activity [61]. Expression level of the PKCθ protein was found to be higher in the duodenum and ileum, compared to colon. This mirrors the expression levels in murine JICC and CICC, as shown in this study (S9A Fig). The functional role of PKCθ in ICC is not known, but a recent study reported that PKCθ protein is hyper-activated in gastrin induced gastrointestinal stromal tumors (GIST) via gastrin and cholecystokinin receptor CCK2R [62], suggesting that the protein promotes ICC proliferation and GIST tumorigenesis.

A gene whose expression has never been ascribed to ICC until this study is *Thbs4* (Fig 4). Various isoforms of *Thbs4* were enriched in JICC and CICC (Fig 4C) and THBS4 protein was detected within ANO1^+^ ICC-MY and ICC-SMP in the colon and ANO1^+^ ICC-MY and ICC-DMP in the jejunum (Fig 4G). The protein was also detected around the ICC, suggesting the protein may be secreted so that it can contribute to the microenvironment in which ICC are maintained in tissues. According to UniProtKB/Swiss-Prot [63], THBS4 is sub-localized to the endoplasmic reticulum and extracellular region. THBS4 is highly expressed in the extracellular region of subventricular zone (SVZ)-generated astrocytes [64]. Its expression was markedly induced by photothrombotic/ischemic cortical injury through binding of the NOTCH1 receptor [64]. *Notch1* has enriched expression in jejunal and colonic PDGFRα^+^ cells when compared to SMC and ICC according to our transcriptome data. PDGFRα^+^ cells express PDGFRα, which acts as a cell-surface receptor for ligands PDGFA-C. THBS4 directly interacts with PDGFA and PDGFB [65]. Since both interstitial cells, PDGFRα^+^ cells and ICC are highly co-localized in colon and jejunal muscles, THBS4 secreted from ICC may regulate the growth of PDGFRα^+^ cells via the NOTCH1 receptor. In the small intestine partial obstruction model, hyperplasia (increase of cell number) of the smooth muscle layer occurs first followed by hypertrophy (increase of cell size) [66]. We have previously reported that SMC are dedifferentiated into highly proliferative PDGFRα^+^ cells, which largely account for smooth muscle hypertrophy in the partial obstruction model [18]. Following partial obstruction, THBS4 was also highly induced in the hypertrophied muscle (unpublished data), suggesting THBS4 is a growth factor that induced the early proliferation of PDGFRα^+^ cells which were dedifferentiated from SMC in the obstructed region. THBS4 protein was decreased in the redifferentiated and hypertrophied SMC as compared to the growing PDGFRα^+^ cells (unpublished data), indicating the protein negatively regulates cell differentiation as well. THBS4 contains four epidermal growth factor (EGF)-like domains, two of which include a Ca^2+^ binding domain (S2 Fig), which are recognized by the receptor integrin α_M_β_2_ [44], suggesting the protein function may be regulated by Ca^2+^ [67]. This protein also binds to heparin [67]. Heparin-binding EGF-like growth factor plays a critical role in both pathological processes (wounding healing, cardiac hypertrophy, tumor progression and metastasis, organ hyperplasia, and atherosclerosis) and normal physiological process (heart development and function) [68, 69]. Taken together, THBS4 may play a key role in the GI smooth muscle development, anatomical positioning of interstitial cells and remodeling under pathological circumstances.

## Supporting information

S1 Fig Confirmation of cell markers expressed in ICC. (A) Expression levels of *Kit* (ICC marker), (B) *Myh11* (SMC marker), (C) *Pdgfra* (PDGFRα^+^ cell marker), and (D) *Uchl1* (PGP9.5, neuronal cell marker) in the jejunum, colon, and isolated JICC and CICC. (E) Expression levels of PDGFRα^+^ cell markers (*Pdgfra*, *Pdgfrb*, *Cd34*, *Kcnn3*, *P2ry1*, and *Ces1d*) and ICC marker (*Kit*) in colon, isolated CICC and CPDGFRα^+^ cells.
(TIF)

S2 Fig Alignment of predicted amino acid sequences from THBS4 transcriptional variants. The open reading frame was identified for each transcriptional variant, and predicted amino acid sequences were aligned together. An N-ternimal signal peptide, Lamin G-like domain, two cell attachment sites (RGD), four EGF-like domains including two calcium binding sites (Ca^2+^), eight thrombospodin 3 (TSP type-3) repeats and C-terminus are annotated. A black arrowhead indicates divisions beween domains.
(TIF)

S3 Fig Identification of potassium, sodium, and cation channel subunits highly and specifically expressed in ICC. (A) Calcium channel isoforms enriched in JICC and CICC. (B) ICC-specific calcium channel isoforms. (C) Potassium channel isoforms enriched in JICC and CICC. (D) ICC-specific potassium channel isoforms. (E) Sodium channel isoforms enriched in JICC and CICC. (F) ICC-specific sodium channel isoforms. (G) Cation channel isoforms enriched in JICC and CICC. (H) ICC-specific cation channel isoforms. Cell specificity was determined by comparative analysis of gene expression profiles among ICC, SMC, and PDGFRα^+^ cells.
(TIF)

S4 Fig Alignment of predicted amino acid sequences from HCN4 transcriptional variants. The open reading frame was identified for each transcriptional variant, and all predicted amino acid sequences were aligned. Six transmembrane helices (S1–S6) and a pore region are shown. Colors on amino acid sequence show distinct regions and segments. Green are start codons found in differentially started variants. Red are positively charged residues in S4 voltage sensing segments. Purple are putative binding sites for cAMP at the cytoplasmic C-terminus.
(DOCX)

S5 Fig Identification of hydrogen and sodium transporter subunits highly and specifically expressed in ICC. (A) Hydrogen transporter isoforms enriched in JICC and CICC. (B) ICC-specific hydrogen transporter isoforms. (C) Sodium transporter isoforms enriched in JICC and CICC. (D) ICC-specific sodium transporter isoforms. Cell specificity was determined by comparative analysis of gene expression profiles among ICC, SMC, and PDGFRα^+^ cells.
(TIF)

S6 Fig Identification of growth factors, receptors, and transcription factors highly and specifically expressed in ICC. (A) Growth factor isoforms enriched in JICC and CICC. (B) ICC-specific growth factor isoforms. (C) Receptor isoforms enriched in JICC and CICC. (D) ICC-specific receptor isoforms. (E) Transcription factor isoforms enriched in JICC and CICC. (F) ICC-specific transcription factor isoforms isoforms. Cell specificity was determined by comparative analysis of gene expression profiles among ICC, SMC, and PDGFRα^+^ cells.
(TIF)

S7 Fig Identification of DNA methylation/demethylation enzymes and methyl-CpG binding proteins highly and specifically expressed in ICC. (A) DNA methyltransferases (*Dnmt1* and *Dnmt3a*), methylcytosine dioxygenases (*Tet1*, *Tet2*, *Tet3*), and DNA oxidative demethylase (*Alkbh1*) enriched in JICC and CICC. (B) ICC-specific isoforms of DNA methylation and demethylation enzymes. (C) Methyl-CpG binding proteins enriched in JICC and CICC. (D) ICC-specific methyl-CpG binding proteins. Cell specificity was determined by comparative analysis of gene expression profiles among ICC, SMC, and PDGFRα^+^ cells.
(TIF)

S8 Fig Identification of histone modifying enzymes highly and specifically expressed in ICC. (A) Histone acetyltransferases enriched in JICC and CICC. (B) ICC-specific histone acetyltransferases. (C) Histone deacetylases enriched in JICC and CICC. (D) ICC-specific histone deacetylases. (E) Histone methyltransferases enriched in JICC and CICC. (F) ICC-specific histone methyltransferases. (G) Histone demethylases enriched in JICC and CICC. (H) ICC-specific histone demethylases. Cell specificity was determined by comparative analysis of gene expression profiles among ICC, SMC, and PDGFRα^+^ cells.
(TIF)

S9 Fig Identification of protein kinases and phosphatases highly and specifically expressed in ICC. (A) Protein kinases enriched in JICC and CICC. (B) ICC-specific protein kinases. (C) Phosphatases enriched in JICC and CICC. (D) ICC-specific phosphatases. Cell specificity was determined by comparative analysis of gene expression profiles among ICC, SMC, and PDGFRα^+^ cells.
(TIF)

S1 Table Summary of ICC transcriptomes obtained from RNA-seq. (XLSX)

S2 Table A list of transcriptional variants expressed in jejunal and colonic ICC. (XLSX)

S3 Table A list of genes expressed in jejunal and colonic ICC. (XLSX)

S4 Table A list of ion channels and transporters expressed in jejunal and colonic ICC. (XLSX)

S5 Table A list of growth factors expressed in jejunal and colonic ICC. (XLSX)

S6 Table A list of receptors expressed in jejunal and colonic ICC. (XLSX)

S7 Table A list of transcription factors expressed in jejunal and colonic ICC. (XLSX)

S8 Table A list of epigenetic enzymes and regulators expressed in jejunal and colonic ICC. (XLSX)

S9 Table A list of protein kinases expressed in jejunal and colonic ICC. (XLSX)

S10 Table A list of phosphatases expressed in jejunal and colonic ICC. (XLSX)

S11 Table Oligonucleotides used in this study. (XLS)
