# Supplementary figures and images for: Transcriptome of interstitial cells of Cajal reveals unique and selective gene signatures
Source: PLoS One. 2017 Apr 20;12(4):e0176031. doi: 10.1371/journal.pone.0176031 (PMC5398589; doi:10.1371/journal.pone.0176031)

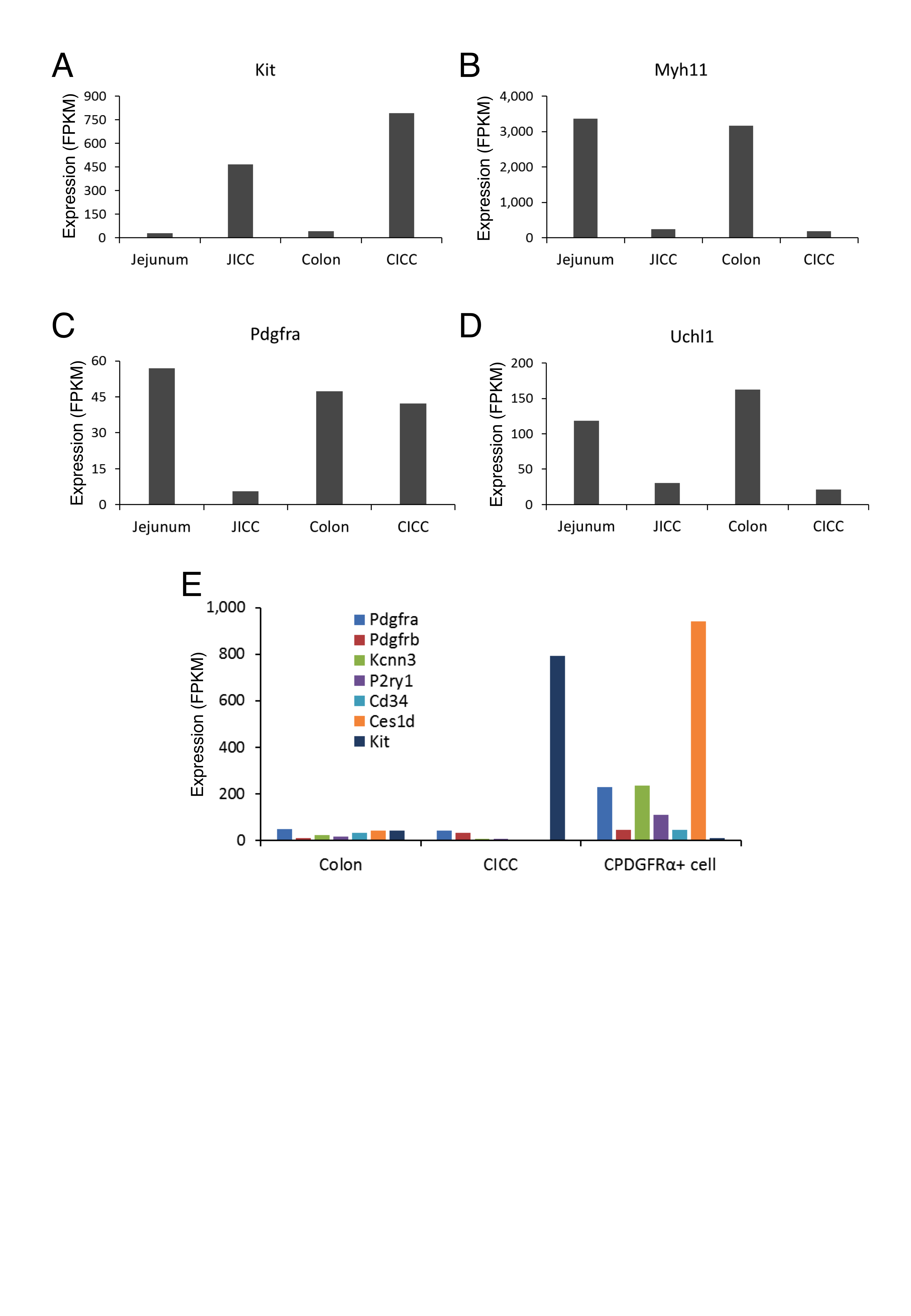

Supplement: S1 Fig — (A) Expression levels of Kit (ICC marker), (B) Myh11 (SMC marker), (C) Pdgfra (PDGFRα+ cell marker), and (D) Uchl1 (PGP9.5, neuronal cell marker) in the jejunum, colon, and isolated JICC and CICC. (E) Expression levels of PDGFRα+ cell markers (Pdgfra, Pdgfrb, Cd34, Kcnn3, P2ry1, and Ces1d) and ICC marker (Kit) in colon, isolated CICC and CPDGFRα+ cells. (TIF) [file pone.0176031.s001.tif]

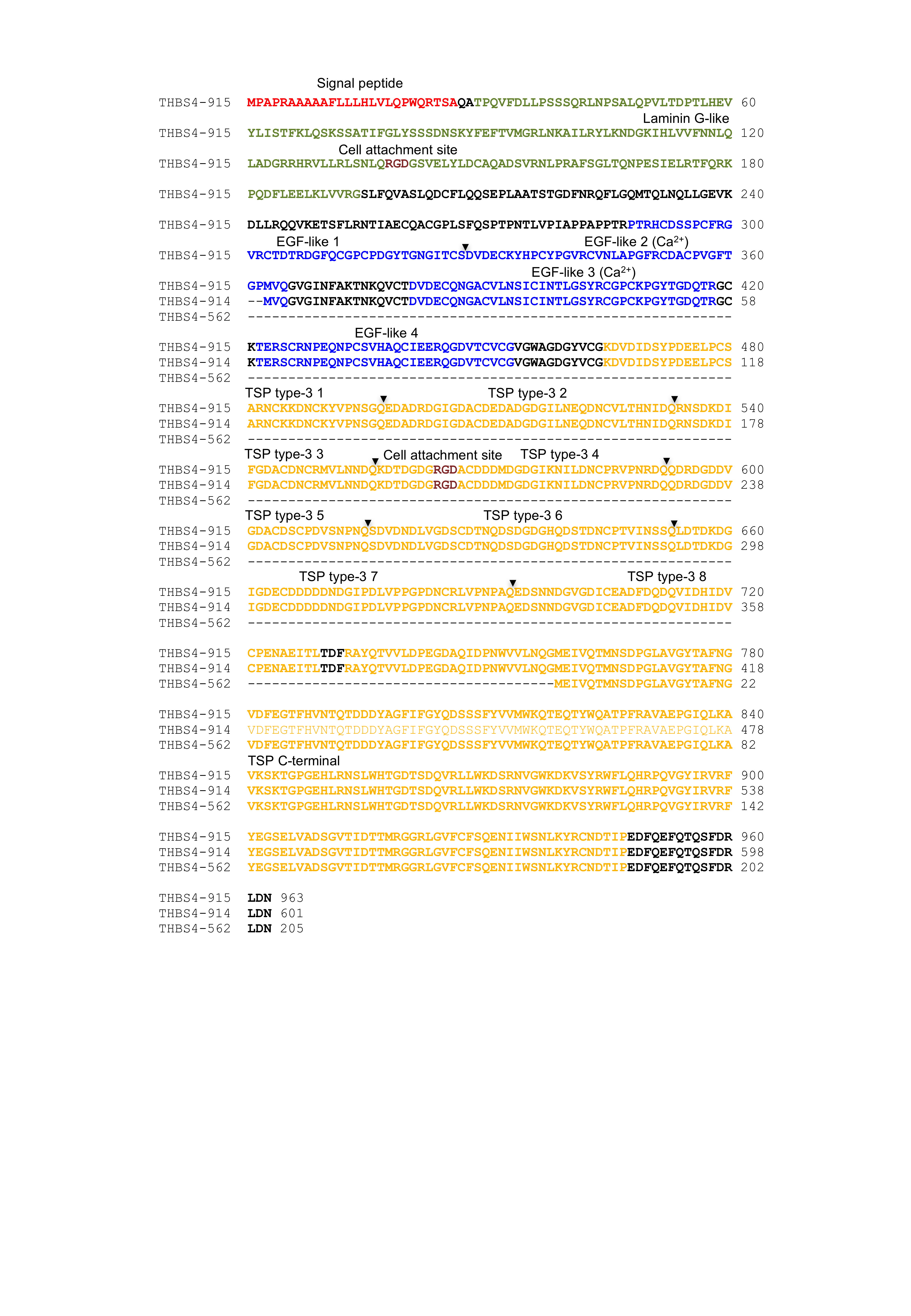

Supplement: S2 Fig — The open reading frame was identified for each transcriptional variant, and predicted amino acid sequences were aligned together. An N-ternimal signal peptide, Lamin G-like domain, two cell attachment sites (RGD), four EGF-like domains including two calcium binding sites (Ca2+), eight thrombospodin 3 (TSP type-3) repeats and C-terminus are annotated. A black arrowhead indicates divisions beween domains. (TIF) [file pone.0176031.s002.tif]

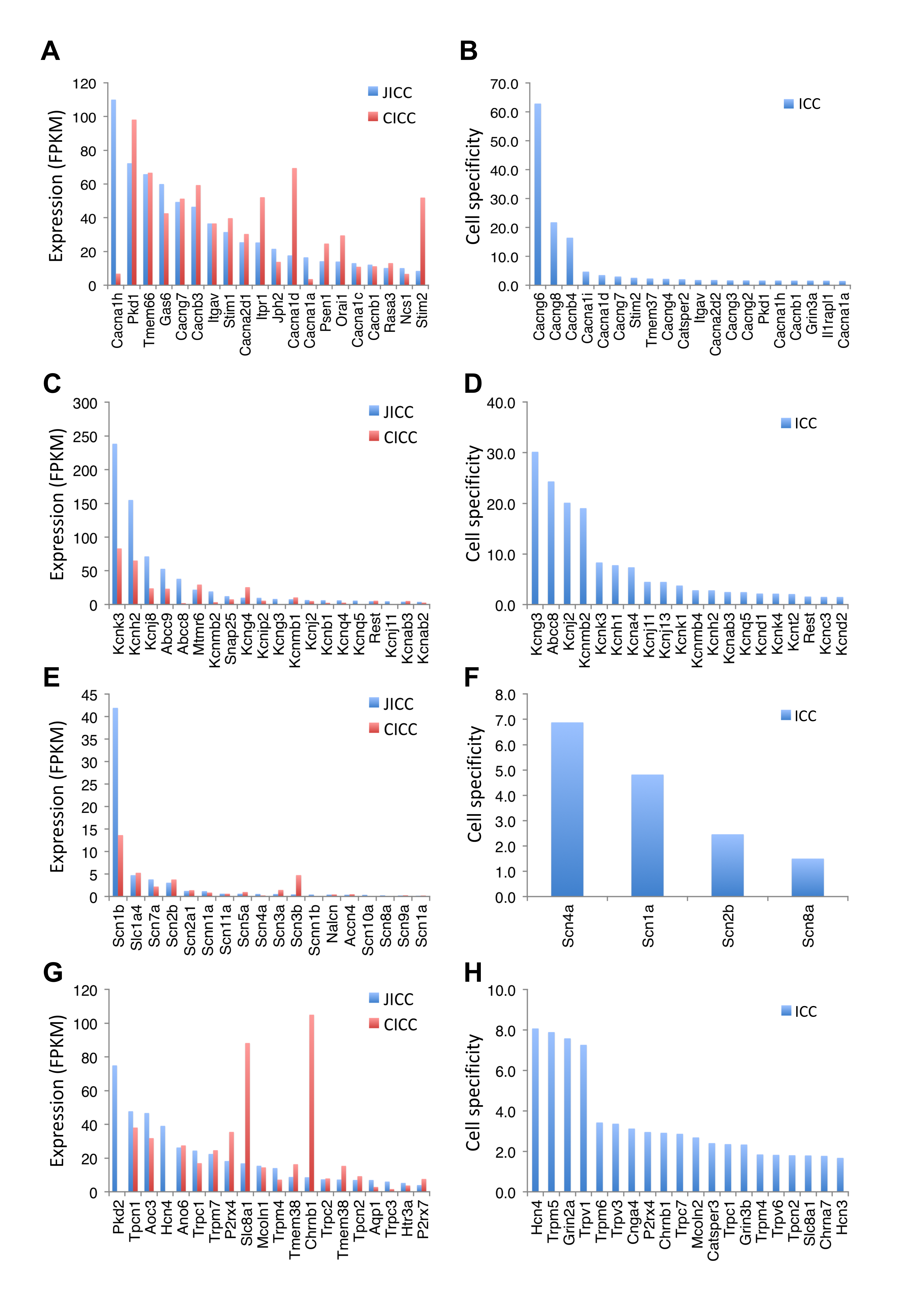

Supplement: S3 Fig — (A) Calcium channel isoforms enriched in JICC and CICC. (B) ICC-specific calcium channel isoforms. (C) Potassium channel isoforms enriched in JICC and CICC. (D) ICC-specific potassium channel isoforms. (E) Sodium channel isoforms enriched in JICC and CICC. (F) ICC-specific sodium channel isoforms. (G) Cation channel isoforms enriched in JICC and CICC. (H) ICC-specific cation channel isoforms. Cell specificity was determined by comparative analysis of gene expression profiles among ICC, SMC, and PDGFRα+ cells. (TIF) [file pone.0176031.s003.tif]

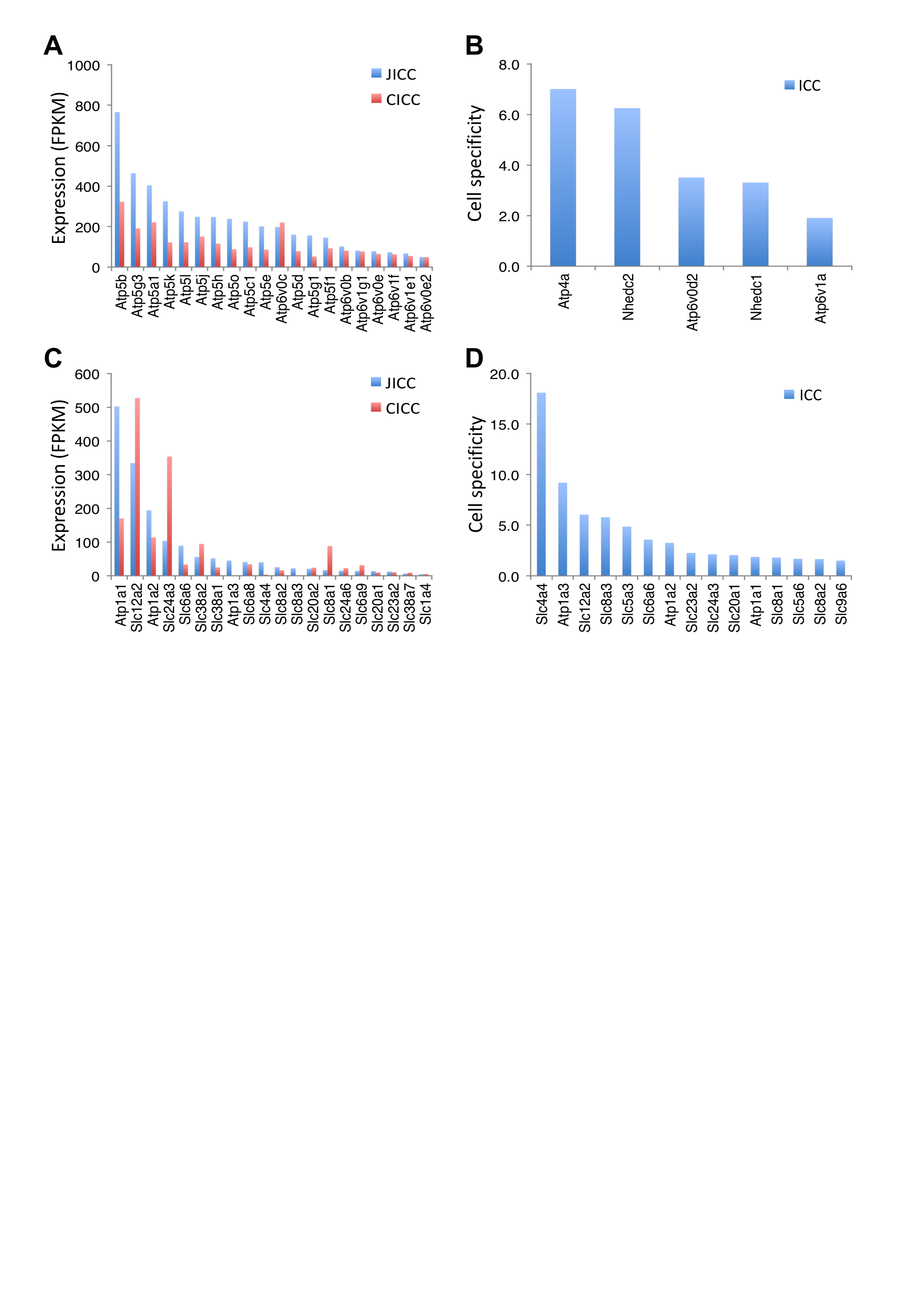

Supplement: S5 Fig — (A) Hydrogen transporter isoforms enriched in JICC and CICC. (B) ICC-specific hydrogen transporter isoforms. (C) Sodium transporter isoforms enriched in JICC and CICC. (D) ICC-specific sodium transporter isoforms. Cell specificity was determined by comparative analysis of gene expression profiles among ICC, SMC, and PDGFRα+ cells. (TIF) [file pone.0176031.s005.tif]

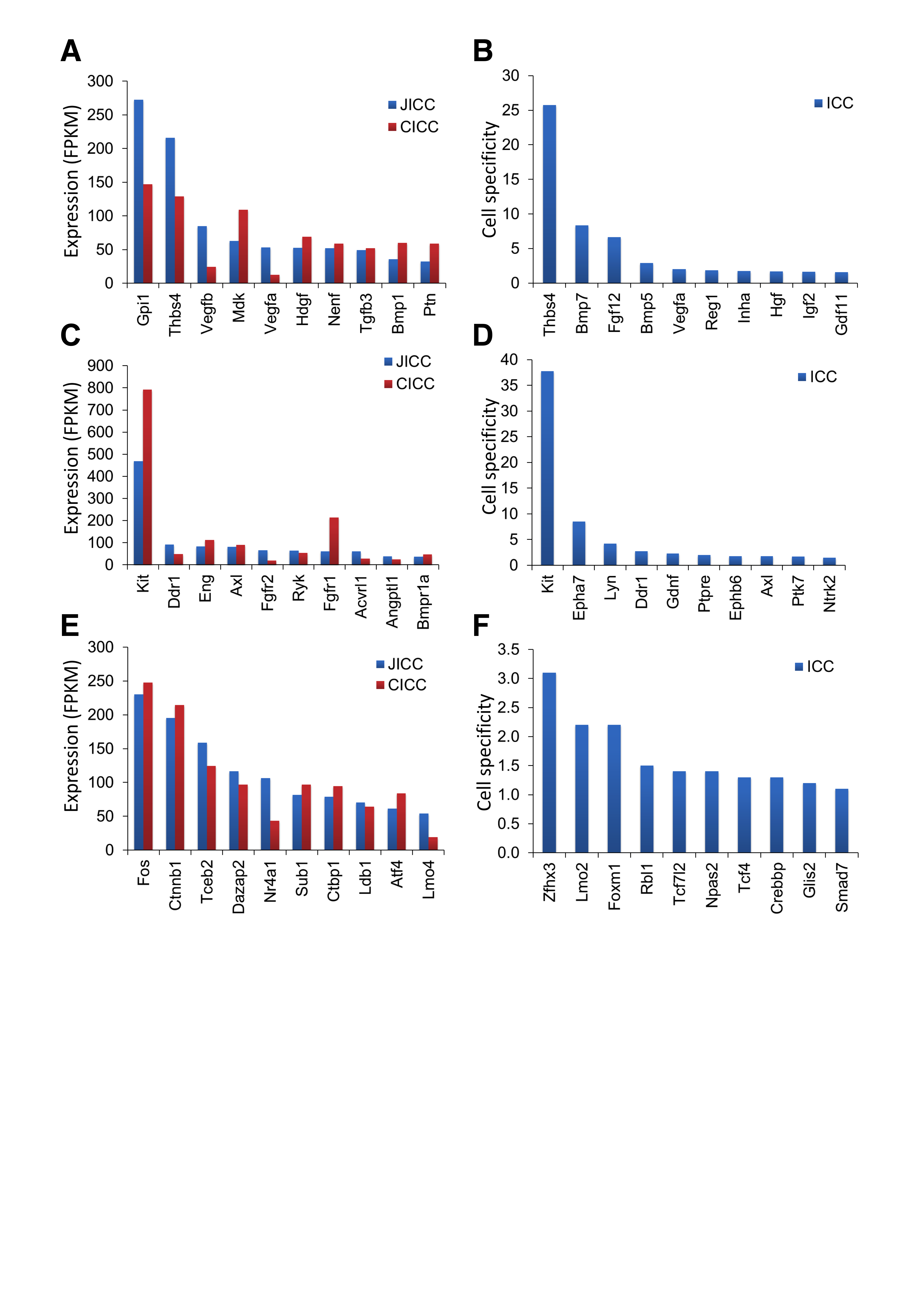

Supplement: S6 Fig — (A) Growth factor isoforms enriched in JICC and CICC. (B) ICC-specific growth factor isoforms. (C) Receptor isoforms enriched in JICC and CICC. (D) ICC-specific receptor isoforms. (E) Transcription factor isoforms enriched in JICC and CICC. (F) ICC-specific transcription factor isoforms isoforms. Cell specificity was determined by comparative analysis of gene expression profiles among ICC, SMC, and PDGFRα+ cells. (TIF) [file pone.0176031.s006.tif]

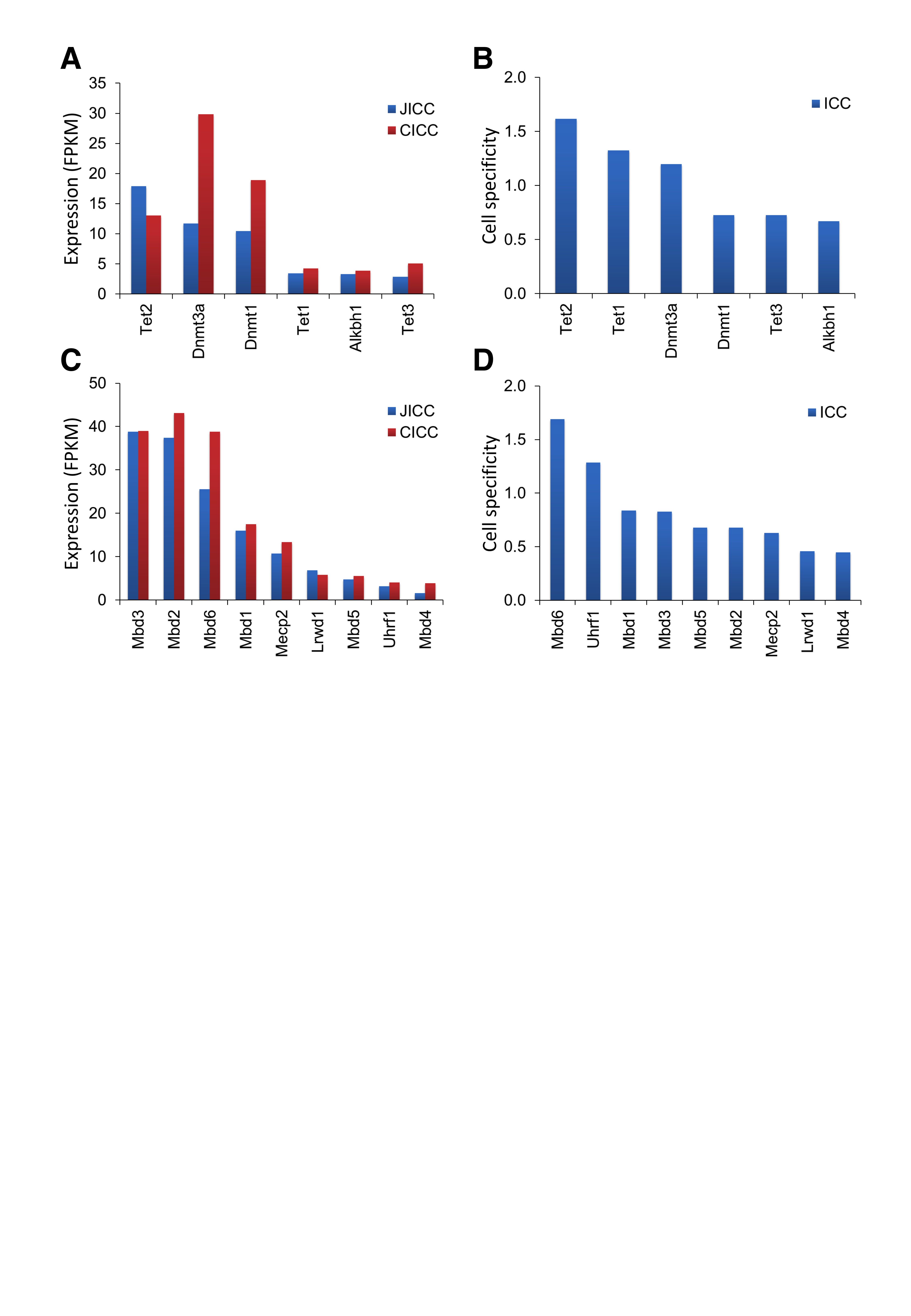

Supplement: S7 Fig — (A) DNA methyltransferases (Dnmt1 and Dnmt3a), methylcytosine dioxygenases (Tet1, Tet2, Tet3), and DNA oxidative demethylase (Alkbh1) enriched in JICC and CICC. (B) ICC-specific isoforms of DNA methylation and demethylation enzymes. (C) Methyl-CpG binding proteins enriched in JICC and CICC. (D) ICC-specific methyl-CpG binding proteins. Cell specificity was determined by comparative analysis of gene expression profiles among ICC, SMC, and PDGFRα+ cells. (TIF) [file pone.0176031.s007.tif]

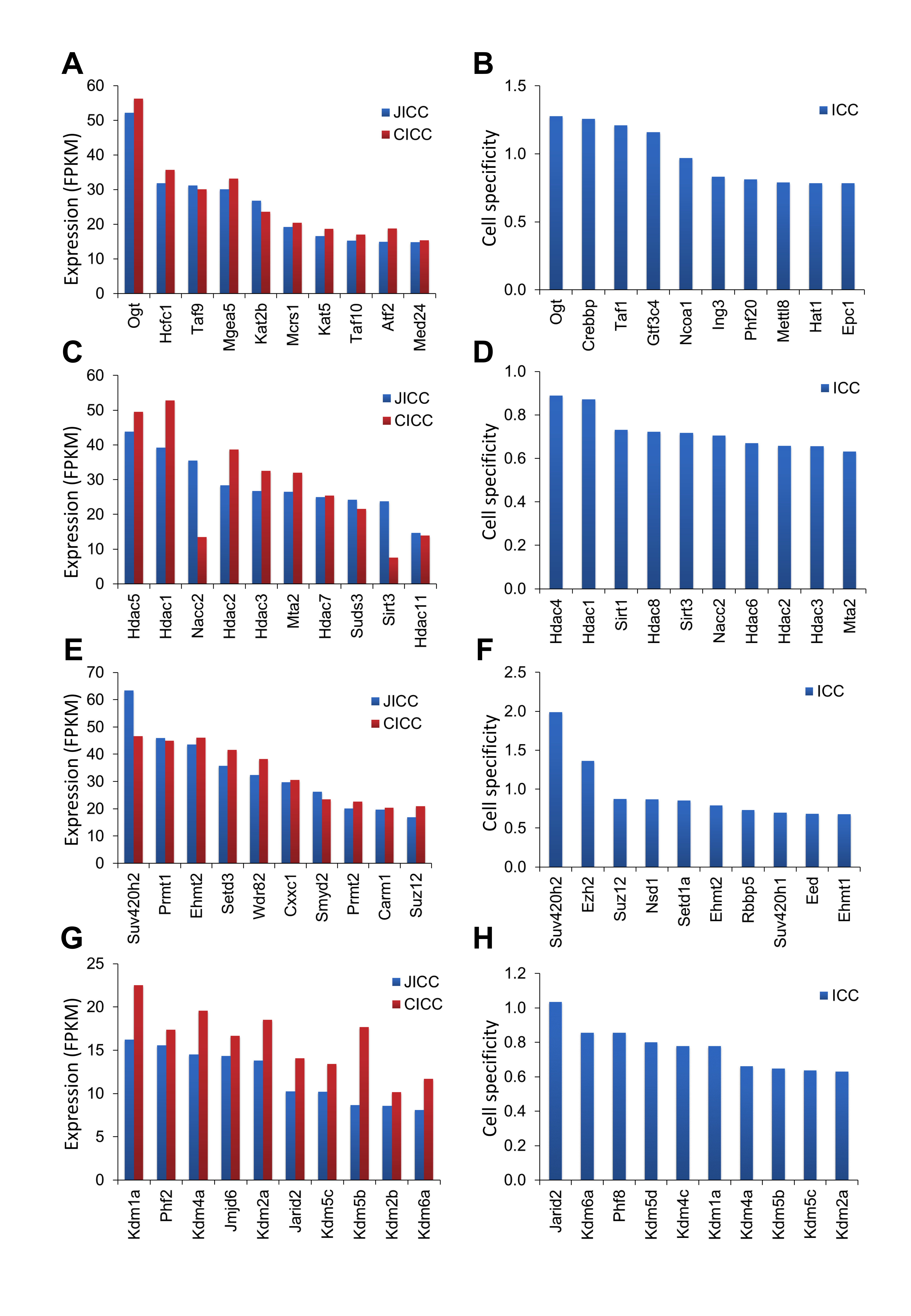

Supplement: S8 Fig — (A) Histone acetyltransferases enriched in JICC and CICC. (B) ICC-specific histone acetyltransferases. (C) Histone deacetylases enriched in JICC and CICC. (D) ICC-specific histone deacetylases. (E) Histone methyltransferases enriched in JICC and CICC. (F) ICC-specific histone methyltransferases. (G) Histone demethylases enriched in JICC and CICC. (H) ICC-specific histone demethylases. Cell specificity was determined by comparative analysis of gene expression profiles among ICC, SMC, and PDGFRα+ cells. (TIF) [file pone.0176031.s008.tif]

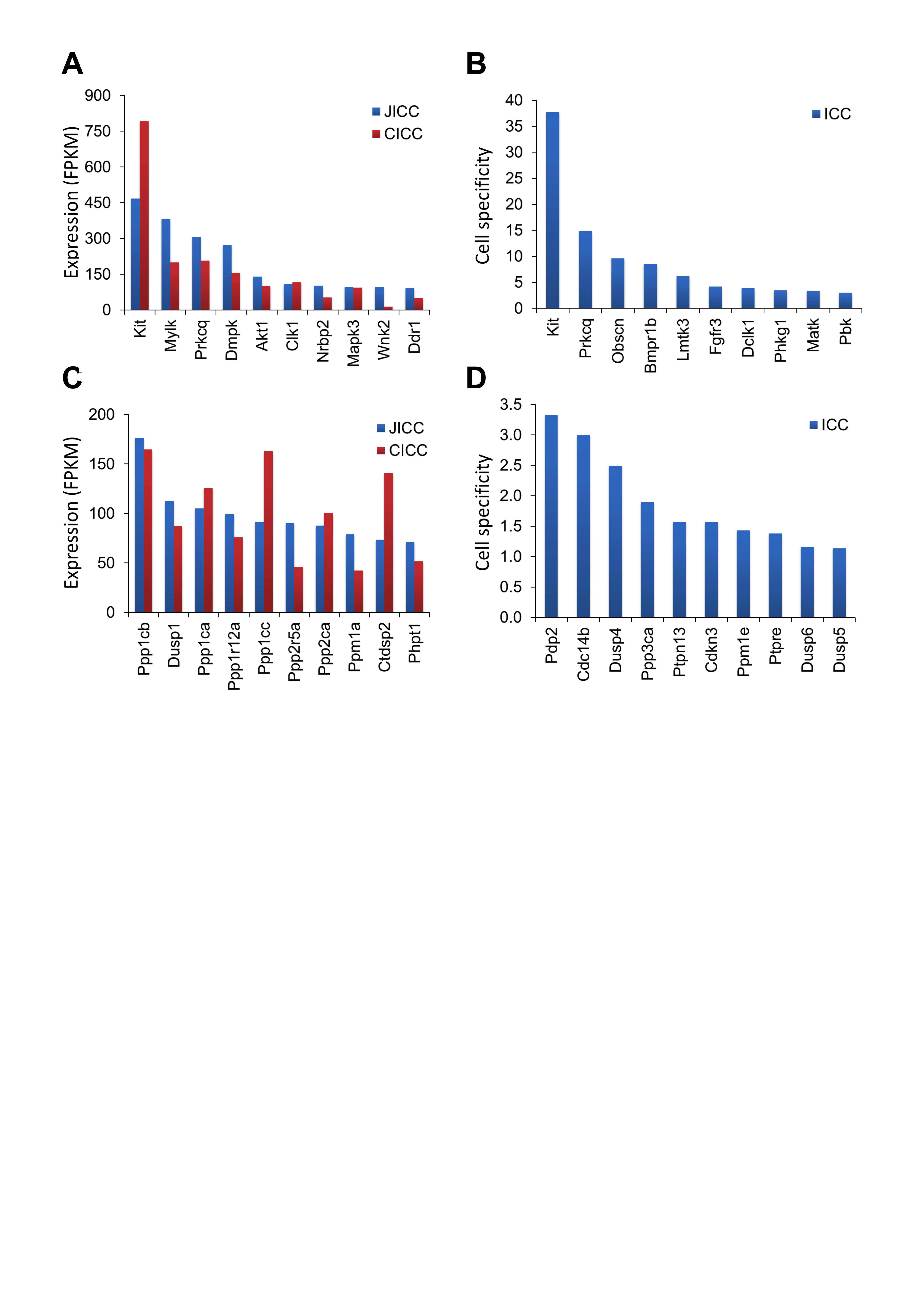

Supplement: S9 Fig — (A) Protein kinases enriched in JICC and CICC. (B) ICC-specific protein kinases. (C) Phosphatases enriched in JICC and CICC. (D) ICC-specific phosphatases. Cell specificity was determined by comparative analysis of gene expression profiles among ICC, SMC, and PDGFRα+ cells. (TIF) [file pone.0176031.s009.tif]
