# Supplementary material for: Transcriptome of interstitial cells of Cajal reveals unique and selective gene signatures
Source: PLoS One. 2017 Apr 20;12(4):e0176031. doi: 10.1371/journal.pone.0176031 (PMC5398589; doi:10.1371/journal.pone.0176031)
Supplement: S4 Fig — The open reading frame was identified for each transcriptional variant, and all predicted amino acid sequences were aligned. Six transmembrane helices (S1–S6) and a pore region are shown. Colors on amino acid sequence show distinct regions and segments. Green are start codons found in differentially started variants. Red are positively charged residues in S4 voltage sensing segments. Purple are putative binding sites for cAMP at the cytoplasmic C-terminus. (DOCX) [file pone.0176031.s004.docx]

HCN4V1 MDKLPPSMRKRLYSLPQQVGAKAWIMDEEEDGEEEGAGGRQDPSRRSIRLRPLPSPSPSV 60

HCN4V1 AAGCSESRGAALGATESEGPGRSAGKSSTNGDCRRFRGSLASLGSRGGGSGGAGGGSSLG 120

Antibody

HCN4V1 HLHDSAEERRLIAAEGDASPGEDRTPPGLATEPERPATAAQPAASPPPQQPPQPASASCE 180

HCN4V1 QPSADTAIKVEGGAAASDQILPEAEVRLGQSGFMQRQFGAMLQPGVNKFSLRMFGSQKAV 240

----------**S1**--------- -------

HCN4V1 EREQERVKSAGFWIIHPYSDFRFYWDLTMLLLMVGNLIIIPVGITFFKDENTTPWIVFNV 300

---**S2**--------- ----------**S3**--------

HCN4V1 VSDTFFLIDLVLNFRTGIVVEDNTEIILDPQRIKMKYLKSWFVVDFISSIPVDYIFLIVE 360

- ----------**S4**---------

HCN4V1 TRIDSEVYKTARALRIVRFTKILSLLRLLRLSRLIRYIHQWEEIFHMTYDLASAVVRIVN 420

HCN4V2 ----------------------------------------------MTYDLASAVVRIVN 14

HCN4V3 ----------------------------------------------MTYDLASAVVRIVN 14

----------**S5**--------- ---------**Pore**---

HCN4V1 LIGMMLLLCHWDGCLQFLVPMLQDFPHDCWVSINGMVNNSWGKQYSYALFKAMSHMLCIG 480

HCN4V2 LIGMMLLLCHWDGCLQFLVPMLQDFPHDCWVSINGMVNNSWGKQYSYALFKAMSHMLCIG 74

HCN4V3 LIGMMLLLCHWDGCLQFLVPMLQDFPHDCWVSINGMVNNSWGKQYSYALFKAMSHMLCIG 74

------ ----------**S6**---------

HCN4V1 YGRQAPVGMSDVWLTMLSMIVGATCYAMFIGHATALIQSLDSSRRQYQEKYKQVEQYMSF 540

HCN4V2 YGRQAPVGMSDVWLTMLSMIVGATCYAMFIGHATALIQSLDSSRRQYQEKYKQVEQYMSF 134

HCN4V3 YGRQAPVGMSDVWLTMLSMIVGATCYAMFIGHATALIQSLDSSRRQYQEKYKQVEQYMSF 134

HCN4V1 HKLPPDTRQRIHDYYEHRYQGKMFDEESILGELSEPLREEIINFNCRKLVASMPLFANAD 600

HCN4V2 HKLPPDTRQRIHDYYEHRYQGKMFDEESILGELSEPLREEIINFNCRKLVASMPLFANAD 194

HCN4V3 HKLPPDTRQRIHDYYEHRYQGKMFDEESILGELSEPLREEIINFNCRKLVASMPLFANAD 194

HCN4V1 PNFVTSMLTKLRFEVFQPGDYIIREGTIGKKMYFIQHGVVSVLTKGNKETKLADGSYFGE 660

HCN4V2 PNFVTSMLTKLRFEVFQPGDYIIREGTIGKKMYFIQHGVVSVLTKGNKETKLADGSYFGE 254

HCN4V3 PNFVTSMLTKLRFEVFQPGDYIIREGTIGKKMYFIQHGVVSVLTKGNKETKLADGSYFGE 254

cAMP cAMP

HCN4V1 ICLLTRGRRTASVRADTYCRLYSLSVDNFNEVLEEYPMMRRAFETVALDRLDRIGKKNSI 720

HCN4V2 ICLLTRGRRTASVRADTYCRLYSLSVDNFNEVLEEYPMMRRAFETVALDRLDRIGKKNSI 314

HCN4V3 ICLLTRGRRTASVRADTYCRLYSLSVDNFNEVLEEYPMMRRAFETVALDRLDRIGKKNSI 314

HCN4V1 LLHKVQHDLNSGVFNYQENEIIQQIVRHDREMAHCAHRVQAAASATPTPTPVIWTPLIQA 780

HCN4V2 LLHKVQHDLNSGVFNYQENEIIQQIVRHDREMAHCAHRVQAAASATPTPTPVIWTPLIQA 374

HCN4V3 LLHKVQHDLNSGVFNYQENEIIQQIVRHDREMAHCAHRVQAAASATPTPTPVIWTPLIQA 374

HCN4V1 PLQAAAATTSVAIALTHHPRLPAAIFRPPPGPGLGNLGAGQTPRHPRRLQSLIPSALGSA 840

HCN4V2 PLQAAAATTSVAIALTHHPRLPAAIFRPPPGPGLGNLGAGQTPRHPRRLQSLIPSALGSA 434

HCN4V3 PLQAAAATTSVAIALTHHPRLPAAIFRPPPGPGLGNLGAGQTPRHPRRLQSLIPSALGSA 434

HCN4V1 SPASSPSQVDTPSSSSFHIQQLAGFSAPPGLSPLLPSSSSSPPPGACGSPPAPTPSTSTA 900

HCN4V2 SPASSPSQVDTPSSSSFHIQQLAGFSAPPGLSPLLPSSSSSPPPGACGSPPAPTPSTSTA 494

HCN4V3 SPASSPSQVDTPSSSSFHIQQLAGFSAPPGLSPLLPSSSSSPPPGACGSPPAPTPSTSTA 494

HCN4V1 AAASTTGFGHFHKALGGSLSSSDSPLLTPLQPGARSPQAAQPPPPLPGARGGLGLLEHFL 960

HCN4V2 AAASTTGFGHFHKALGGSLSSSDSPLLTPLQPGARSPQAAQPPPPLPGARGGLGLLEHFL 554

HCN4V3 AAASTTGFGHFHKALGGSLSSSDSPLLTPLQPGARSPQAAQPPPPLPGARGGLGLLEHFL 554

HCN4V1 PPPPSSRSPSSSPGQLGQPPGELSLGLAAGPSSTPETPPRPERPSFMAGASGGASPVAFT 1020

HCN4V2 PPPPSSRSPSSSPGQLGQPPGELSLGLAAGPSSTPETPPRPERPSFMAGASGGASPVAFT 614

HCN4V3 PPPPSSRSPSSSPGQLGQPPGELSLGLAAGPSSTPETPPRPERPSFMAGASGGASPVAFT 614

HCN4V1 PRGGLSPPGHSPGPPRTFPSAPPRASGSHGSLLLPPASSPPPPQVPQRRGTPPLTPGRLT 1080

HCN4V2 PRGGLSPPGHSPGPPRTFPSAPPRASGSHGSLLLPPASSPPPPQVPQRRGTPPLTPGRLT 674

HCN4V3 PRGGLSPPGHSPGPPRTFPSAPPRASGSHGSLLLPPASSPPPPQVPQRRGTPPLTPGRLT 674

HCN4V1 QDLKLISASQPALPQDGAQTLRRASPHSSGESVAAFSLYPRAGGGSGSSGGLGPPGRPYG 1140

HCN4V2 QDLKLISASQPALPQDGAQTLRRASPHSSGESVAAFSLYPRAGGGSGSSGGLGPPGRPYG 734

HCN4V3 QDLKLISASQPALPQDGAQTLRRASPHSSGESVAAFSLYPRAGGGSGSSGGLGPPGRPYG 734

HCN4V1 AIPGQHVTLPRKTSSGSLPPPLSLFGARAASSGGPPLTTAAPQREPGARSEPVRSKLPSN 1200

HCN4V2 AIPGQHVTLPRKTSSGSLPPPLSLFGARAASSGGPPLTTAAPQREPGARSEPVRSKLPSN 794

HCN4V3 AIPGQHVTLPRKTSSGSLPPPLSLFGARAASSGGPPLTTAAPQREPGARSEPVRSKLPSN 794

HCN4V1 L 1201

HCN4V2 L 795

HCN4V3 L 795
